# RSPO2 promotes progression of ovarian cancer through dual receptor-mediated FAK/Src signaling activation

**DOI:** 10.1016/j.isci.2022.105184

**Published:** 2022-09-23

**Authors:** Rulu Pan, Yan Yu, Haiyan Zhu, Wenyi Zhang, Yuan Qin, Lin Ye, Juji Dai, Ren Huang, Xinyan Peng, Siqi Ye, Ziqi Lin, Shishun Huang, Shuyi Chong, Liting Lu, Xincheng Lu

**Affiliations:** 1 School of Basic Medical Sciences, Wenzhou Medical University, Wenzhou 325035, China; 2 Clinical Laboratory, Yiwu Traditional Chinese Medicine Hospital, Yiwu 322000, China; 3 Department of Gynecology, Shanghai First Maternity and Infant Hospital, Tongji University School of Medicine, Shanghai 200126, China; 4 Department of Radiotherapy, The First Affiliated Hospital of Wenzhou Medical University, Wenzhou 325000, China; 5 Department of Colorectal and Anal Surgery, The First Affiliated Hospital of Wenzhou Medical University, Wenzhou 325000, China

**Keywords:** Cell biology, cancer

## Abstract

R-spondin 2 (RSPO2) drives the potentiation of Wnt signaling and is implicated in tumorigenesis in multiple cancers, but its role in ovarian cancer has not been investigated. Here, we reported that RSPO2 promoted the growth and metastasis of ovarian cancer through the activation of FAK/Src signaling cascades. RSPO2 enhanced the autophosphorylation of FAK and Src through a unique dual receptors mechanism. First, RSPO2-LGR4 interaction prevented the endocytic degradation of LGR4 and promoted LGR4-mediated translocation of Src to the plasma membrane. Second, RSPO2 directly bound to integrin β3 as a ligand and enhanced the stability of integrins, and both actions potentiated autoactivation of FAK and/or Src in ovarian cancer cells. RSPO2 expression was increased in ovarian tumors and was associated with poor prognosis in patients. Our study highlights the importance of RSPO2 in ovarian tumor progression and suggests that targeting RSPO2/FAK/Src cascades may constitute potential approaches to inhibit the progression of aggressive ovarian cancer.

## Highlights

• RSPO2 plays an oncogenic role in ovarian cancer progression
• RSPO2 acts as a FAK/Src activator instead of as a Wnt agonist in ovarian cancer
• RSPO2 promotes the LGR4-mediated Src autoactivation
• RSPO2 binds to integrin β3 as a ligand to potentiate the activation of FAK

## Graphical abstract

**Figure:**
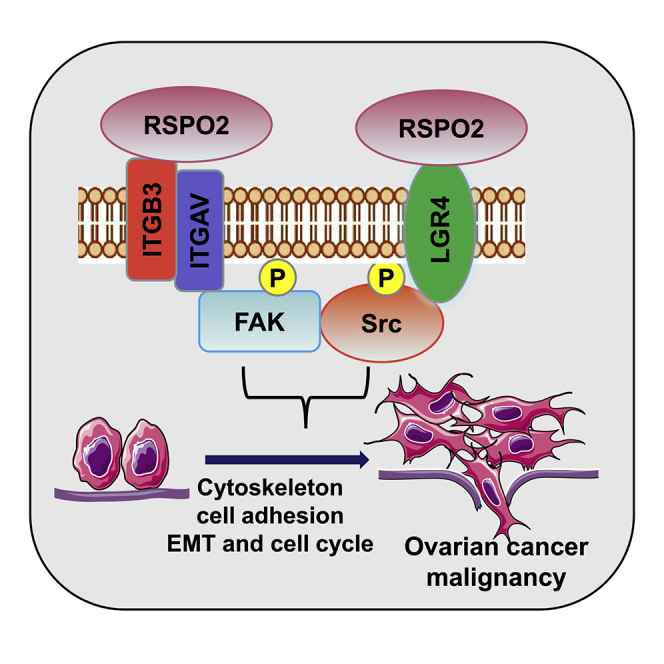

## Introduction

Ovarian cancer, with epithelial ovarian cancer (EOC) as the most common histological type, is the most lethal gynecological malignancy, causing 184,799 deaths annually (Bray et al., 2018). Owing to its late-stage diagnosis, peritoneal dissemination, high relapse rate, and acquisition of drug resistance, EOC has a poor prognosis, with a 5-year survival rate of approximately 30% (Reid et al., 2017; Lheureux et al., 2019). Therefore, characterizing novel therapeutic targets and molecular mechanisms that mediate tumor progression is of great clinical significance to improve the treatment of ovarian cancer.

The R-spondin (RSPO) protein family has four homologous members that are evolutionarily conserved in vertebrates, showing 60% amino acid sequence homology (Jin and Yoon, 2012). All four RSPOs contain tandem furin-like repeats (FUs), a thrombospondin type 1 (TSP) domain, and a basic region (BR) (Jin and Yoon, 2012; Yoon and Lee, 2012). RSPOs were first identified as potent agonists of the Wnt/β-catenin signaling pathway (Kim et al., 2008; Nam et al., 2006). Mechanistically, RSPOs bind to three leucine-rich repeat-containing G-protein-coupled receptors (LGRs), LGR4/5/6, through their FUs domain. RSPO/LGR interactions neutralize two transmembrane E3 ubiquitin ligases, ZNRF3 and RNF43, which prevents the internalization and degradation of cell surface Wnt receptors and eventually amplifies Wnt/β-catenin signaling (Carmon et al., 2011; Hao et al., 2012). RSPO-mediated Wnt/β-catenin signaling activation participates in a broad range of biological and physiological processes, such as cell proliferation, development, stem cell maintenance, and tumorigenesis (Yan et al., 2017; Vidal et al., 2020; Knight and Hankenson, 2014; Ter Steege and Bakker, 2021; Kim et al., 2005). R-spondin 2 (RSPO2) is an important member of the RSPO family. In humans, polymorphisms in the RSPO2 gene lead to genetic susceptibility to Dupuytren contracture (Dolmans et al., 2011). Mice with the knockout of Rspo2 exhibit severe developmental abnormalities, including lung hypoplasia and defects in craniofacial and limb development (Szenker-Ravi et al., 2018; Bell et al., 2008). RSPO2 has also been implicated in hair growth, osteoblastic differentiation, and osteoarthritis through the potentiation of Wnt/β-catenin signaling (Knight et al., 2018; Smith et al., 2016; Okura et al., 2019). Notably, RSPO2 has recently been identified as an important regulator of tumorigenicity. Rearrangement and fusion of RSPO2 occur in several types of tumors, such as colon, prostate, and liver tumors (Seshagiri et al., 2012; Longerich et al., 2019; Robinson et al., 2015). RSPO2-mediated activation of Wnt/β-catenin signaling has been shown to promote the growth and metastasis of diverse tumors, including tongue squamous cell carcinomas, pancreatic and breast tumors (Ilmer et al., 2015; Ter Steege and Bakker, 2021; Zhang et al., 2019). Intriguingly, two previous studies demonstrated that RSPO2 inhibits colorectal cancer growth through unique LGR5-mediated Wnt/β-catenin signaling negative feedback mechanism, and RSPO2-mediated suppression of noncanonical Wnt signaling also exerted an inhibitory effect on colon tumor metastasis (Wu et al., 2014; Dong et al., 2017). A recent study showed that RSPO2 acts as a tumor suppressor in HCC by inhibiting the MAPK signaling pathway (Zheng et al., 2020). Taken together, these findings suggest that the role of RSPO2 in tumorigenesis is complex and that its functions may be dependent on the type of cancer, the presence of receptors, and the cellular context.

RSPO2 signaling has been shown to be essential for oocyte-driven intercellular communication and follicular growth (Cheng et al., 2013; De Cian et al., 2020). Moreover, the mutation of RSPO2 is associated with primary ovarian insufficiency and the survival of patients with high-grade serous ovarian cancer (Bouilly et al., 2017; Lee et al., 2020). These findings imply that RSPO2 may play a role in female fertility, ovarian follicle maturation, and even ovarian carcinogenesis. However, the biological function of RSPO2 in ovarian cancer progression remains unexplored. In this study, we investigated the role of RSPO2 in ovarian cancer progression. We demonstrated that RSPO2 enhanced the malignant biological behaviors of ovarian cancer cells, including proliferation, migration, and invasion, as well as their ability to adhere to the extracellular matrix *in vitro*. Furthermore, we showed that RSPO2 promoted the metastatic spread of cancer cells in an orthotopic ovarian xenograft model and that these phenotypes were primarily ascribed to the activation of FAK/Src signaling pathways. Mechanistically, we revealed novel crosstalk between RSPO2 and FAK/Src signaling and determined that RSPO2 can mediate the autoactivation of FAK and Src by binding to two specific receptors.

## Results

### RSPO2 expression is elevated in ovarian cancer

**Figure 1:**
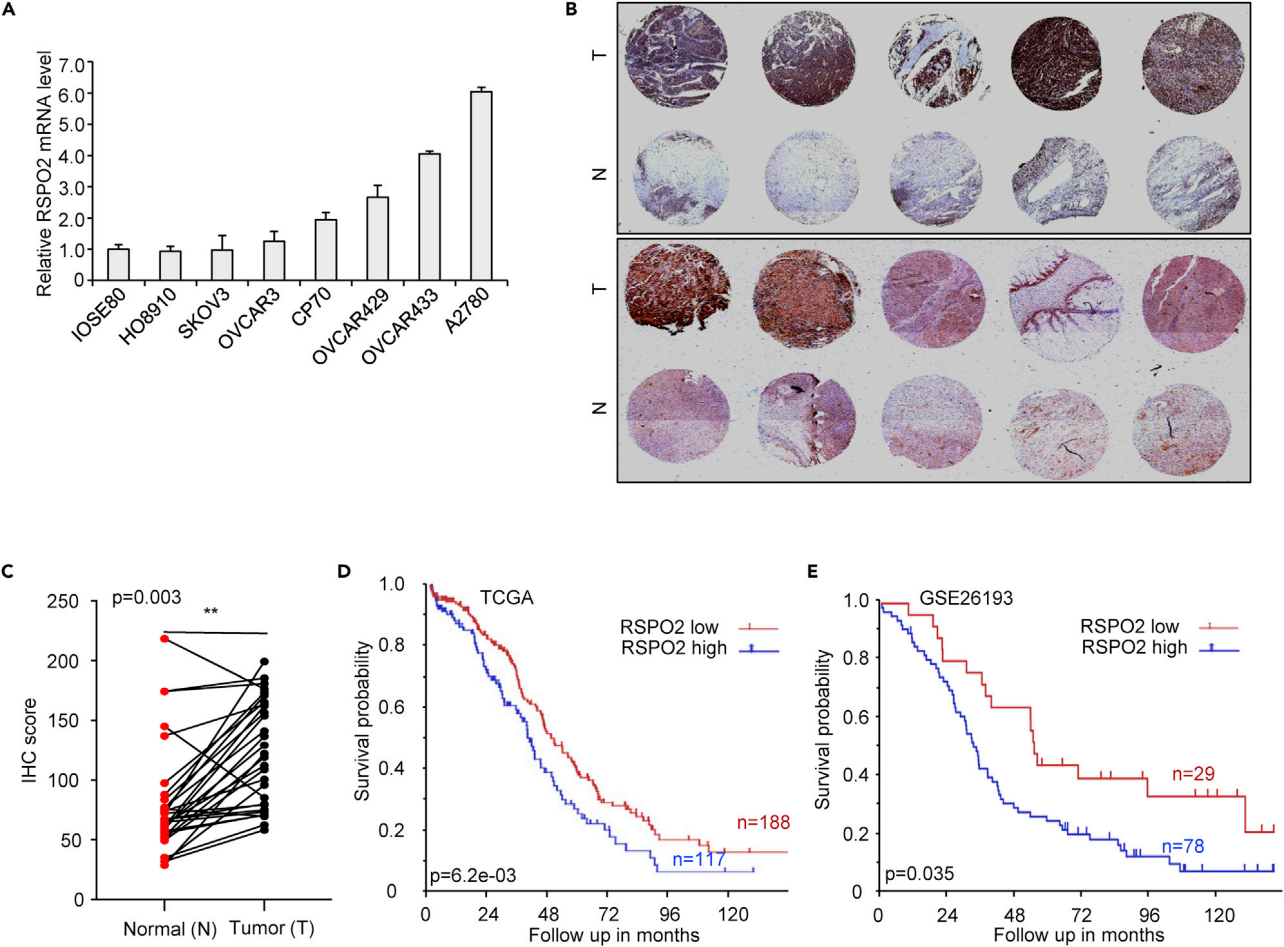
Expression and pathological significance of RSPO2 in ovarian cancer (A) Relative mRNA expression levels of RSPO2 in ovarian cancer cell lines. RSPO2 mRNA levels were determined using RT-qPCR. mRNA expression levels were normalized to those in normal ovarian epithelial cells (IOSE80). (B) Representative images of immunohistochemical staining for RSPO2 in a tissue microarray containing thirty ovarian tumors (T) and paired normal ovarian (N) tissues. Scale bar, 50 μm. (C) Immunohistochemical staining score for RSPO2 in the tissue microarray (n = 30). ∗∗p < 0.01, calculated by two-tailed unpaired t-test. (D) Kaplan-Meier survival analysis of ovarian cancer patients with RSPO2-high vs. RSPO2-low tumors from the TCGA cohort. (E) Kaplan-Meier survival analysis of ovarian cancer patients with RSPO2-high vs. RSPO2-low tumors from the GSE26193 cohort.

To determine whether the expression of RSPO2 is dysregulated in ovarian cancer, we first examined the expression of RSPO2 in multiple ovarian cancer cell lines by qRT-PCR. The mRNA expression of RSPO2 in cancer cells was generally higher than that in ovarian epithelial IOSE80 cells (Figure 1A). Next, we measured RSPO2 protein expression in ovarian cancer specimens by tissue microarray analysis. The microarray test set comprised 30 patients newly diagnosed with EOC and aged 27 to 84 years (mean, 55 years). Immunohistochemical analysis of paired tumor and nontumor tissue specimens showed that RSPO2 was more intensely and extensively expressed in tumor tissues than in normal tissues (Figure 1B). Staining quantification showed that RSPO2 expression in ovarian tumor tissues was significantly higher than that detected in the paired normal tissues (Figure 1C). Moreover, mining of the ovarian cancer cohort in the TCGA database showed that high RSPO2 expression was associated with a lower survival rate in patients with ovarian cancer (Figure 1D). Kaplan-Meier analysis of another ovarian cancer dataset (GSE26193) also showed a significant association between high RSPO2 expression and poor survival (Figure 1E). Collectively, these results suggest that the expression of RSPO2 is elevated in ovarian cancer and associated with poor prognosis in patients.

### RSPO2 promotes ovarian cancer growth and metastasis

**Figure 2:**
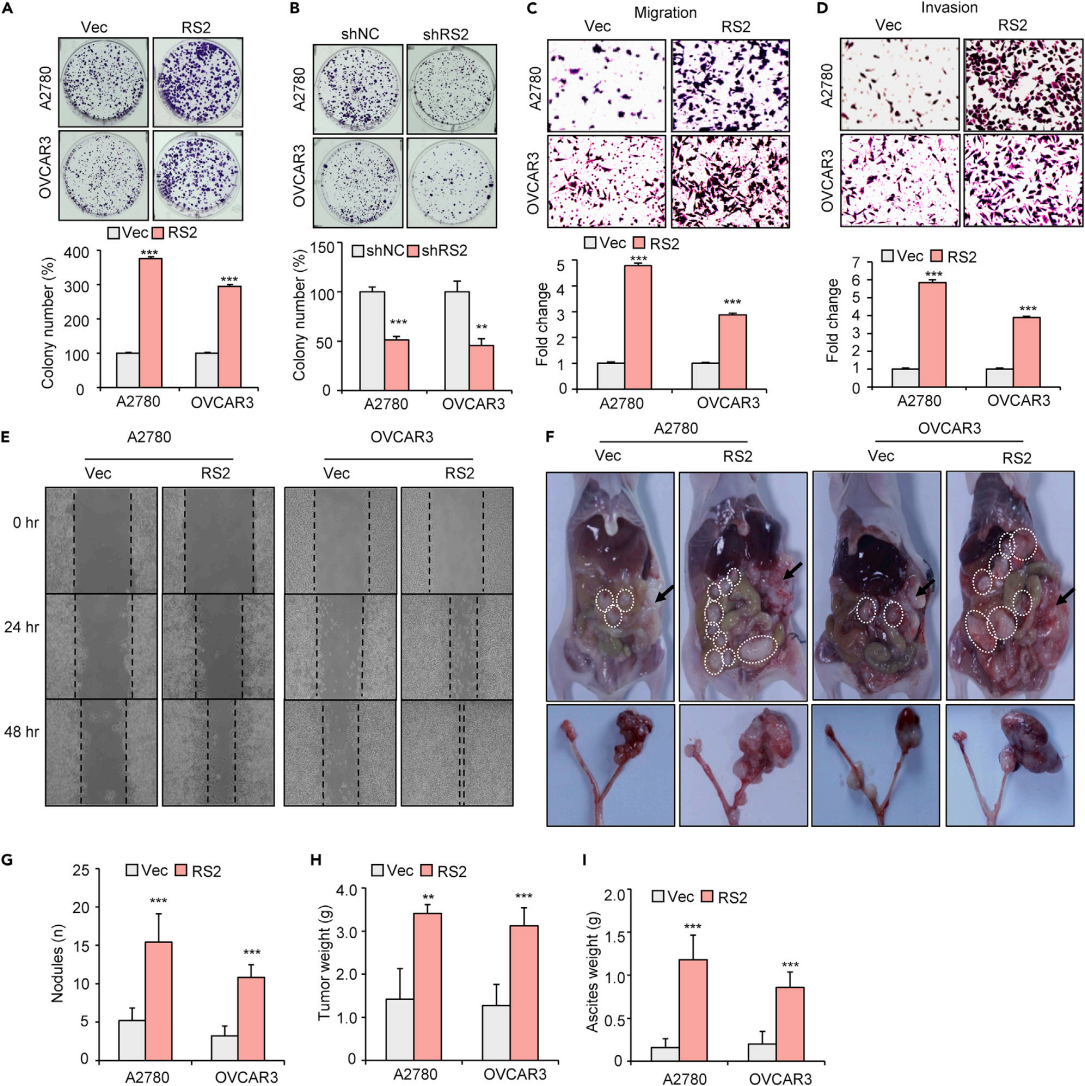
RSPO2 promotes ovarian cancer growth and metastasis (A) Representative images (upper panel) and quantification (lower panel) of the colony formation results of A2780 and OVCAR3 cells after RSPO2 overexpression. Cells stably overexpressing RSPO2 (RS2, pooled) or empty vector (Vec) were used. ∗∗∗p < 0.001 vs. Vec; two-tailed Student’s t-test. Error bars indicate mean ± SD. (B) Colony formation assay in A2780 and OVCAR3 cells after shRNA-mediated RSPO2 knockdown. The lentiviral vector containing shRNA against RSPO2 (shRS2) was designed using the most effective interference sequence, siRS2-2#. ∗∗p < 0.01 and ∗∗∗p < 0.001 vs. control (shNC); two-tailed Student’s t-test. Error bars indicate mean ± SD. (C and D) Representative images (upper panel) and quantification (lower panel) of the Transwell assay results show that the overexpression of RSPO2 enhanced the migration (C) and invasion (D) of ovarian cancer cells. (E) Wound healing assay showing increased wound closure in the RSPO2-overexpressing cell group. (F) Representative images of tumor nodules in the peritoneum (upper panel) and ovary (lower panel) in the orthotopic mouse model. A2780 and OVCAR3 cells stably overexpressing RSPO2 (RS2, pooled) or empty vector (Vec) were inoculated into the ovary (n = 5 per group). The white dashed circles indicate the metastatic nodules and the black arrow shows the tumor formed in ovarian *in situ*. (G–I) Disseminated tumor nodules and ascites fluid in the peritoneal cavity were harvested and measured after mice were sacrificed. The total number of nodules (G), aggregate tumor weight (H), and ascites weight (I) were calculated for each mouse. ∗∗p < 0.01 and ∗∗∗p < 0.001 vs. Vec; two-tailed Student’s t-test. Error bars indicate mean ± SD.

The dysregulated expression of RSPO2 in tumor tissues inspired us to investigate its role in ovarian malignancy. Based on the characteristic that RSPO2 is a secretory protein, we first established stable transfectants of RSPO2-overexpressing ovarian tumor cells by pooling G418-resistant clones (Figure S1A). Overexpression of RSPO2 in A2780 and OVCAR3 tumor cells markedly enhanced their growth capability as evaluated by MTT and colony formation assays (Figures 2A and S1B). To determine the specificity of RSPO2 on tumor growth, we next screened two siRNAs (siRS2-1# and siRS2-2#) that can effectively target RSPO2 in two ovarian cancer cell lines, and then used the most effective interference sequence siRS2-2# to construct the lentiviral vector (shRS2) (Figures S1C and S1D). siRNA and shRNA-mediated RSPO2 knockdown both significantly reduced cell growth of A2780 and OVCAR3 tumor cells (Figures 2B, S1E, and S1F). Together, these results indicate that RSPO2 plays a promotive role in ovarian cancer cell growth *in vitro*. Peritoneal dissemination and metastasis are characteristics of ovarian malignancy (Yeung et al., 2015). Ectopic overexpression of RSPO2 resulted in increases of 4- to 6-fold in the migration and invasion abilities of A2780 and OVCAR3 cells (Figures 2C and 2D). RSPO2 overexpression also increased motility in both ovarian cancer cell lines (Figure 2E). In contrast, knockdown of RSPO2 markedly suppressed the migration and invasion of A2780 and OVCAR3 tumor cells (Figures S2A and S2B). Consistent with the migration-promoting function of RSPO2 *in vitro*, stable overexpression of RSPO2 markedly promoted metastatic colonization of ovarian cancer cells in an orthotopic mouse model, including increases in the number of metastatic nodules, tumor size, and tumor weight (Figures 2F–2H and S2E). Mice injected with RSPO2-overexpressing cells also showed greater accumulation of ascites fluid in the peritoneal cavity than did control mice (Figure 2I). In addition, mice injected subcutaneously with A2780 cells (with a high level of RSPO2 expression) harboring shRSPO2 developed a markedly decreased tumor burden compared to that in mice injected with shNC control cells (Figures S2C and S2D). Taken together, these results strongly suggest that RSPO2 functions as an oncogene in ovarian cancer progression by promoting tumor cell growth and metastasis.

### RSPO2 promotes epithelial-mesenchymal transition and cell cycle progression in ovarian cancer cells

**Figure 3:**
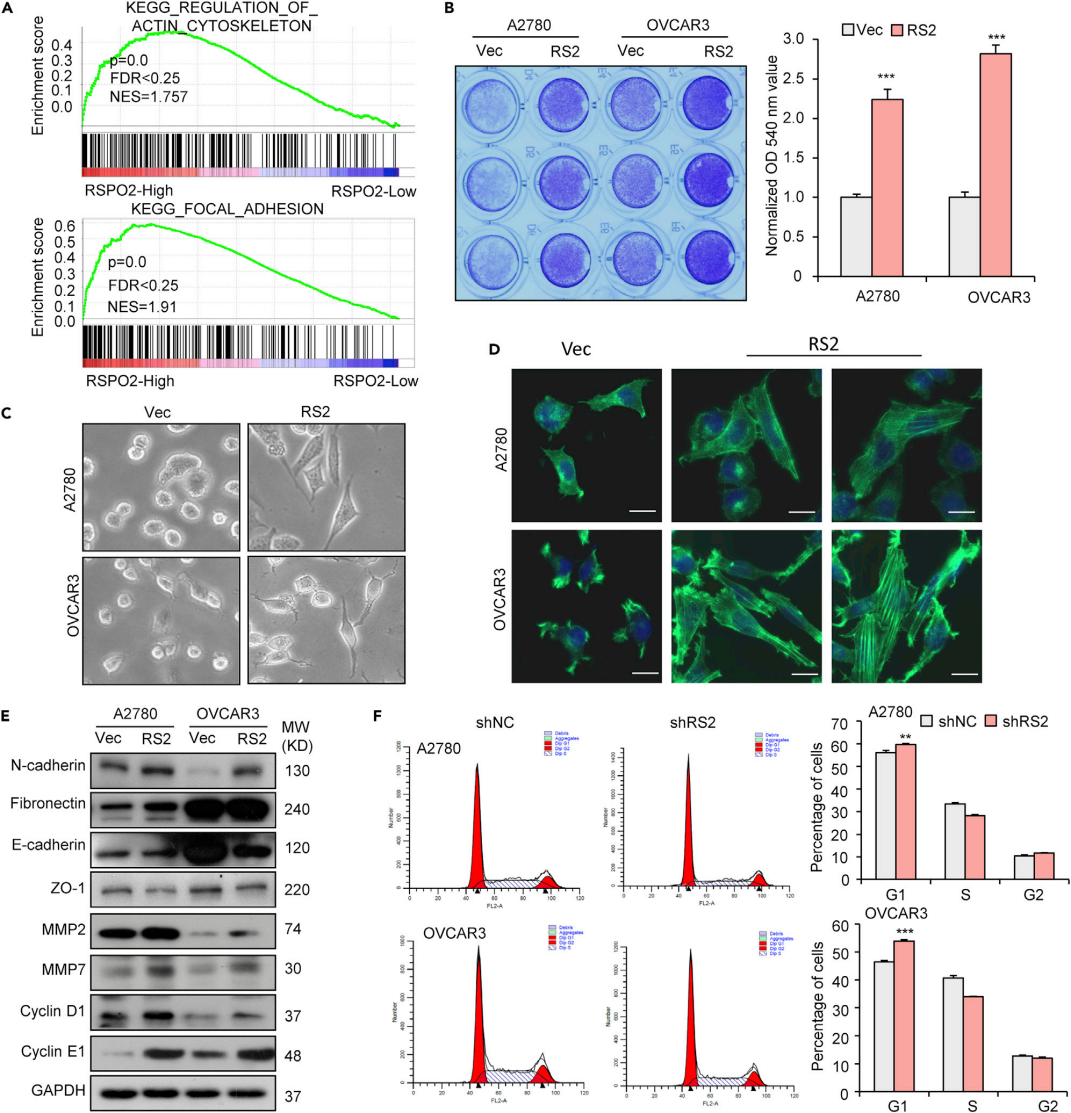
Effects of RSPO2 on ovarian cancer cell adhesion, EMT, and cell cycle progression (A) GSEA of the TCGA ovarian cancer cohort showing that pathways associated with the cytoskeleton and focal adhesion were enriched in tumors with high RSPO2 expression. TCGA dataset was separated into two groups based on the mRNA expression of RSPO2. The expression cutoff for KEGG analysis is first (RSPO2-High) and last quartile (RSPO2-Low) of mRNA expression of RSPO2. (B) Effect of RSPO2 on cell adhesion. Quantitation of adhesion and cell attachment, as evaluated by cellular binding to vitronectin, was performed using vector control cells (Vec) and cells stably overexpressing RSPO2 (RS2). ∗∗∗p < 0.001 vs. Vec. (C) Representative light micrographs showing that stable overexpression of RSPO2 altered the morphology of ovarian cancer cells (magnification, 200×). (D) Phalloidin staining showing actin filaments in A2780 and OVCAR3 cells stably overexpressing RSPO2. Scale bar, 20 μm. (E) Western blot analysis of EMT and cell cycle markers in cells stably overexpressing RSPO2. (F) Flow cytometric analysis of the cell cycle in RSPO2-silenced ovarian cancer cells. Cells were infected with lentivirus containing shRS2 or nontargeting shNC for 48 h. The data shown are the mean ± SD of a representative experiment performed in triplicate (lower panel). ∗∗p < 0.01 and p∗∗∗ <0.001 vs. shNC; two-tailed Student’s t-test. Error bars indicate mean ± SD.

Gene set enrichment analysis (GSEA) and Kyoto Encyclopedia of Genes and Genomes (KEGG) pathway analysis of the TCGA database showed that genes composing the actin cytoskeleton and focal adhesion program signatures, two programs associated with metastatic properties of cancer cells, were highly enriched in ovarian tumor samples with high RSPO2 expression (Figure 3A). Consistent with the bioinformatic analysis results, overexpression of RSPO2 led to increased adhesion of A2780 and OVCAR3 cells to the extracellular matrix component in culture (Figure 3B). Morphologically, overexpression of RSPO2 promoted the transformation of most ovarian cancer cells from a round (epithelial phenotype) to a spindle shape (mesenchymal phenotype) (Figure 3C). F-actin staining further confirmed RSPO2-mediated cytoskeletal rearrangement, including the gain of actin stress fibers and increased lamellipodia and filopodia formation (Figure 3D). As the reorganization of the actin cytoskeleton and concomitant formation of membrane protrusions are essential steps in cell migration during the activation of the epithelial-mesenchymal transition (EMT) program (Nieto et al., 2016), we next assessed the effect of RSPO2 on protein markers associated with EMT. In RSPO2-overexpressing A2780 and OVCAR3 cells, the expression levels of N-cadherin and fibronectin were increased, whereas those of E-cadherin and ZO-1 were markedly reduced (Figure 3E). In addition, RSPO2 overexpression induced the upregulation of matrix metalloproteinases (MMPs), including MMP2 and MMP7, in both cell lines (Figure 3E). These data suggest that RSPO2 affects the EMT process and extracellular matrix degradation, both of which play a significant role in the invasion of ovarian cancer cells. Overexpression of RSPO2 also elicited elevated expression of cell cycle proteins such as cyclin D1 and E1 (Figure 3E). Correspondingly, the knockdown of RSPO2 triggered G1 arrest (Figure 3F). These data indicate that RSPO2 is involved in cell cycle regulation. Taken together, the above results suggest that RSPO2 promotes ovarian cancer progression by affecting cell adhesion, EMT, and cell cycle progression.

### RSPO2 promotes ovarian cancer progression via FAK/Src signaling activation

**Figure 4:**
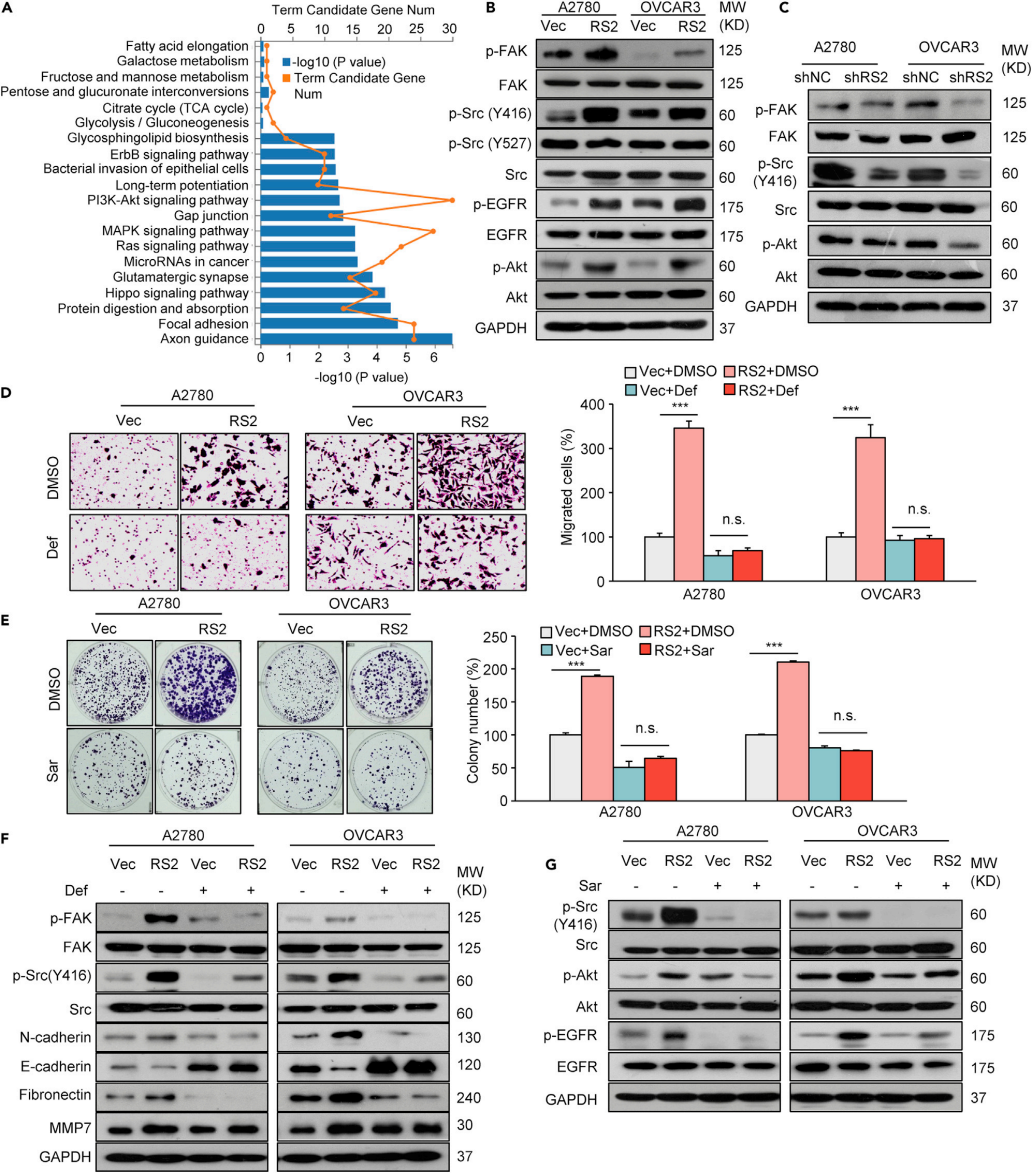
RSPO2 promotes ovarian cancer growth and metastasis through FAK/Src signaling cascades (A) KEGG enrichment analysis of the pathways affected by overexpression of RSPO2 in A2780 cells. (B) Effects of RSPO2 overexpression on the phosphorylation of FAK (Tyr397), Src (Tyr416, Y416), Src (Tyr527, Y527), EGFR (Tyr1068), and Akt (Ser473). Proteins were extracted from A2780 and OVCAR3 cells stably overexpressing RSPO2 (RS2, pooled) or empty vector (Vec) and were analyzed by Western blotting. (C) The effects of RSPO2 knockdown on the phosphorylation of FAK, Src, and Akt were analyzed by Western blotting. Cells were infected with lentivirus containing shRS2 or nontargeting shNC for 48 h. (D) Representative images (left panel) and quantification (right panel) of Transwell assay results showing that the FAK inhibitor defactinib (Def) abolished the promotive effect of RSPO2 overexpression on cell migration. Cells were treated with DMSO or 1.0 μM defactinib for 3 h. (E) Representative images (left panel) and quantification (right panel) of colony formation assay results showing that the Src inhibitor saracatinib (Sar) abolished the promotive effect of RSPO2 overexpression on cell proliferation. Cells were treated with DMSO or saracatinib (Sar, 10 μM) for 1 h. (F) Defactinib abolished the effect of RSPO2 on the expression of EMT markers and MMP7. A2780 and OVCAR3 cells stably overexpressing RSPO2 (RS2, pooled) or empty vector (Vec) were treated with DMSO or 1.0 μM defactinib for 3 h. (G) Saracatinib blocked RSPO2-induced EGFR/Akt phosphorylation. Cells were treated with DMSO or saracatinib (Sar, 10 μM) for 24 h n.s., not significant; p∗∗∗ <0.001; two-tailed Student’s *t* test. Error bars indicate mean ± SD.

RSPO2 promotes a variety of biological processes involved in tumor progression by potentiating Wnt/β-catenin signaling (Yoon and Lee, 2012; Ter Steege and Bakker, 2021). Surprisingly, our initial experiments showed that overexpression of RSPO2 or treatment with recombinant RSPO2 protein did not stimulate obvious Wnt/β-catenin signaling responsiveness in A2780 and OVCAR3 cells (Figures S3A–S3D). Manipulating the expression of RSPO2 did not change the levels of Frizzled 6/7, nor did the Frizzled inhibitor niclosamide abolish the tumor-promoting effect of RSPO2 in two cells (Figures S3E–S3H). To elucidate the Wnt-independent mechanisms by which RSPO2 promotes ovarian cancer progression, we compared the gene expression profiles of parental and RSPO2-overexpressing A2780 cells through transcriptomic sequencing. Similar to the findings in the previous KEGG analysis using a TCGA cohort, the dysregulated genes were enriched in cytoskeletal/focal adhesion-related signatures. Tumor growth- and cell survival-related pathways, such as MAPK and PI3K/Akt, were profoundly enriched and upregulated in RSPO2-overexpressing A2780 cells (Figure 4A). Consistent with the transcriptome analysis results, either overexpression of RSPO2 or treatment with recombinant RSPO2 protein enhanced the phosphorylation of FAK at Tyr397 (p-FAK), Src at Tyr416 (p-Src, Y416), EGFR at Tyr1068 (p-EGFR) and Akt at Ser473 (p-Akt) without affecting the abundance of the corresponding proteins (Figures 4B, S4A, and S4B). Markedly increased FAK/Src/Akt phosphorylation was also observed in RSPO2-high metastases from xenograft tumors (Figure S4C). Conversely, RSPO2-depleted cells showed lower p-FAK, p-Src and p-Akt levels than the nontargeting control-transduced cells (Figure 4C). Furthermore, treatment with Src and FAK inhibitors abrogated the promotive effect of RSPO2 on the proliferation and the migration of ovarian cancer cells (Figures 4D, 4E, S4D, and S4E). These results suggest that the activation of FAK and Src is responsible for RSPO2-promoted ovarian cancer progression. In addition, the inhibition of FAK activity abolished the effect of RSPO2 on EMT marker and MMP7 expression, supporting its critical role in cell migration and invasion (Figure 4F). Inhibition of Src activity attenuated RSPO2-induced EGFR/Akt phosphorylation (Figure 4G), whereas the suppression of Akt activity largely reduced the promotive effect of RSPO2 on cell proliferation (Figures S4F–S4G), suggesting that RSPO2-induced Src/Akt activation mainly accounts for tumor growth. Taken together, these sets of data suggest that RSPO2 promotes ovarian cancer progression through the activation of FAK/Src signaling cascades.

### RSPO2 stimulates the autoactivation of Src via the LGR4 receptor

**Figure 5:**
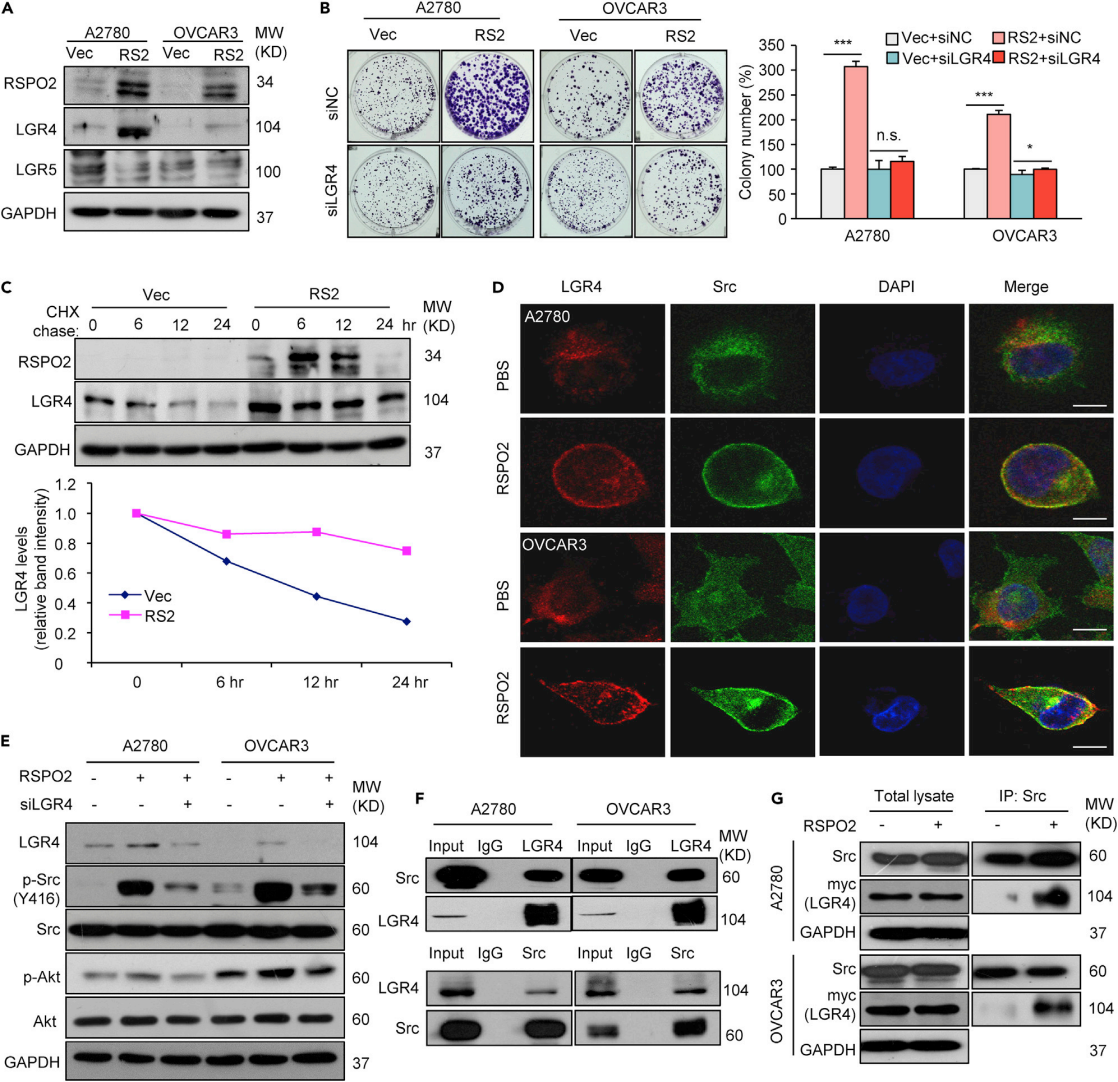
RSPO2 promotes the plasma membrane translocation and autophosphorylation of Src via the LGR4 receptor (A) Western blot analysis of LGR4 and LGR5 in ovarian cancer cells stably overexpressing RSPO2 (RS2, pooled) or vector control (Vec). (B) Representative images (left panel) and quantification (right panel) of colony formation assay results showing that knockdown of LGR4 diminished RSPO2-induced cell growth. A2780 and OVCAR3 cells stably overexpressing RSPO2 (RS2, pooled) or empty vector (Vec) were transfected with siRNA against LGR4 (siLGR4) or a scrambled nontargeting sequence (siNC), and a colony formation assay was then performed. n.s., not significant; ∗p < 0.05 and p∗∗∗ <0.001; two-tailed Student’s *t* test. Error bars indicate mean ± SD. (C) Cycloheximide (CHX)-chase assay to determine the stability of LGR4 in ovarian cancer cells. The LGR4 protein level was measured by Western blotting. A2780 cells without (Vec) or with RSPO2 expression were treated with CHX (500 nM) for the times indicated. Quantification of the LGR4 protein levels was normalized to the GAPDH and zero-time control data. (D) RSPO2 treatment enhanced the plasma membrane distribution of LGR4 and Src in A2780 and OVCAR3 cells. Colocalization of LGR4 with Src was analyzed by fluorescence confocal microscopy. Cells were treated with PBS or RSPO2 protein (200 ng/mL) at 4°C for 2 h. Scale bar, 10 μm. (E) Presilencing of LGR4 attenuated RSPO2-induced Src/Akt phosphorylation. Cells pretransfected with siRNA against LGR4 (siLGR4) or siNC were treated with PBS control or recombinant RSPO2 protein (200 ng/mL) for 12 h. (F) Co-IP analysis of the interaction between endogenous LGR4 and Src in A2780 and OVCAR3 cells. (G) RSPO2 protein treatment enhanced the interaction of Src and LGR4 in A2780 and OVCAR3 cells overexpressing LGR4. Cells transiently transfected with the myc-tagged LGR4 plasmid were treated with 200 ng/mL RSPO2 protein for 12 h, and cell lysates were collected for Co-IP.

Next, we investigated the molecular mechanisms by which RSPO2 activates FAK/Src signaling cascades in ovarian tumor cells. As RSPOs often directly bind to their receptors, LGRs, to potentiate downstream signaling pathways (Yoon and Lee, 2012), we first examined the changes in LGRs expression in response to RSPO2 stimulation. Western blot analysis showed that either ectopic RSPO2 overexpression or RSPO2 protein treatment increased the protein level of LGR4 but had no significant effect on the level of LGR5 (Figures 5A and S5A). Upregulated LGR4 protein expression was also confirmed in xenograft tumor samples with high RSPO2 expression (Figure S5B). Moreover, knockdown of LGR4 in A2780 and OVCAR3 cells largely antagonized the growth promotive effect of RSPO2 and partially impaired the RSPO2-induced cancer cell migration (Figures 5B, S5C, and S5D). These results suggest that the upregulation of LGR4 plays a key role in RSPO2-promoted ovarian cancer growth. RSPO2 did not affect the mRNA level of LGR4 in either of these ovarian cancer cell lines (Figure S5E). However, cycloheximide (CHX)-chase assay showed that overexpression of RSPO2 markedly delayed the degradation of LGR4 protein (Figure 5C). Moreover, dense colocalization of LGR4 and LAMP1 was observed in the cytoplasm of A2780 and OVCAR3 cells, whereas RSPO2 protein treatment attenuated the colocalization of these two proteins and significantly increased the distribution of LGR4 on the plasma membrane (Figure S5F). Taken together; these results suggest that RSPO2 prevents the endocytosis and lysosome-mediated degradation of LGR4 in ovarian cancer cells. Presilencing of LGR4 impaired RSPO2-stimulated Src/Akt activation (Figure 5E). In addition, ectopic overexpression of LGR4 enhanced Src phosphorylation in A2780 and OVCAR3 cells, whereas concomitant overexpression of LGR4 and RSPO2 further sensitized cells to Src activation induced by RSPO2 or LGR4 alone (Figures S6A and S6B). Collectively, these results suggest that LGR4 plays a key role in RSPO2-induced Src activation. Immunofluorescence imaging showed that Src was diffusely expressed throughout the cytoplasm in A2780 and OVCAR3 cells and that RSPO2 protein treatment caused its translocation from the cytosol to the plasma membrane (Figure 5D), which is the characteristic of Src autophosphorylation (Roskoski, 2015). Notably, significantly increased the colocalization of LGR4 and Src was observed on the plasma membrane of RSPO2-treated cells (Figure 5D). These results prompted us to consider that RSPO2 may promote membrane translocation and autophosphorylation of Src through the LGR4-Src interaction. Indeed, ectopically expressed RSPO2 pulled down endogenous LGR4 or Src in both A2780 and OVCAR3 cells (Figure S6C), and reciprocal interactions between endogenous LGR4 and Src were validated by Co-IP (Figure 5F). Furthermore, RSPO2 protein treatment obviously increased the affinity of LGR4 for Src (Figure 5G). Taken together; these results suggest that RSPO2, LGR4, and Src can form a complex on the plasma membrane and that RSPO2 stimulates the autoactivation of Src through LGR4-mediated membrane translocation of Src.

### RSPO2 potentiates FAK signaling via binding to integrin receptors

**Figure 6:**
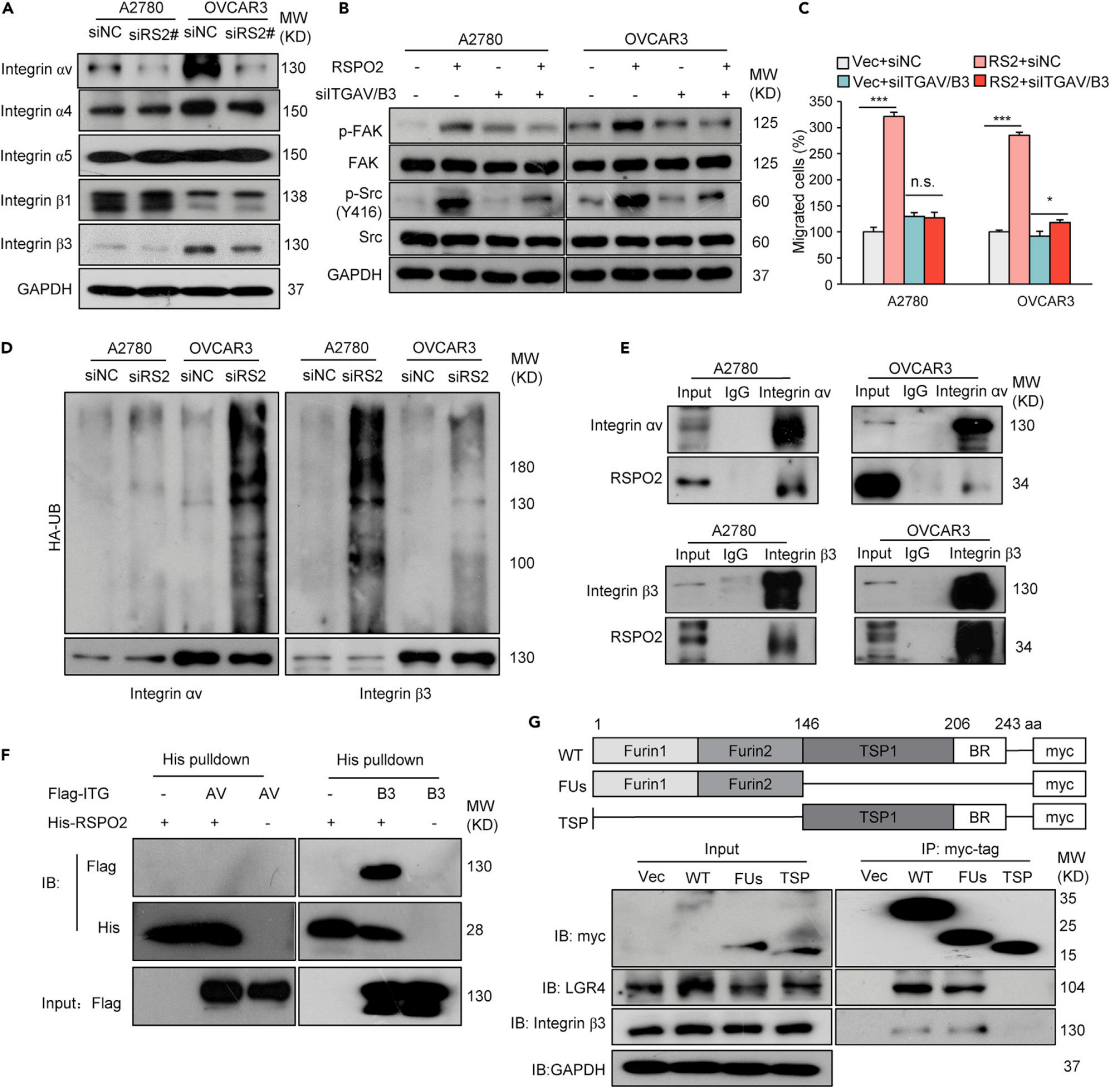
RSPO2 potentiates FAK phosphorylation by binding to integrin β3 (A) Alterations in integrin expression in A2780 and OVCAR3 cells following RSPO2 depletion were analyzed by Western blotting. Cells were transfected with a scrambled nontargeting sequence (siNC) or siRNA against RSPO2 (siRS2#). (B) Presilencing of integrin αv/β3 impaired the enhancing effect of RSPO2 on FAK and Src phosphorylation. Cells pretransfected with siRNAs against both integrin αv and β3 (siITGAV/B3) or siNC (−) were treated with recombinant RSPO2 protein (200 ng/mL) for 12 h. (C) Transwell assay results showing that the silencing of integrin αv/β3 blocked RSPO2-induced cell migration. A2780 and OVCAR3 cells stably overexpressing RSPO2 (RS2, pooled) or empty vector (Vec) were transfected with siRNAs against integrin αv and β3 (siITGAV/B3) or siNC and were then subjected to a Transwell assay. n.s., not significant; ∗∗∗p < 0.001; two-tailed Student’s *t* test. Error bars indicate mean ± SD. (D) Effects of RSPO2 knockdown on the ubiquitination levels of integrin αv and β3 in A2780 and OVCAR3 cells. (E) Co-IP analysis of the interactions between endogenous RSPO2 and integrin αv and β3 in A2780 and OVCAR3 cells. (F) An *in vitro* pulldown assay was performed to validate the interaction between RSPO2 and integrin subunits. Recombinant His-tagged RSPO2 protein was used to pull down Flag-tagged integrin-αv or -β3 from HEK293T cell lysates. (G) Co-IP analysis of the interactions between RSPO2 mutants and LGR4 or ITGB3. OVCAR3 cells were transfected with empty vector (Vec), myc-tagged RSPO2 (WT) or mutants containing only two furin domains (FUs) or a TSP-1 domain (TSP), and cell lysates were immunoprecipitated and probed with anti-Myc, anti-LGR4, or anti-integrin-β3 antibodies. Upper panel, schematic diagram of the RSPO2 domain structure and truncation mutants.

Integrins are well-established upstream inducers of FAK/Src signaling activation (Mitra and Schlaepfer, 2006). In a small-scale screen, we found that knockdown of RSPO2 decreased endogenous integrin αv and β3 levels in A2780 and OVCAR3 cells (Figure 6A). Correspondingly, stable overexpression of RSPO2 elevated the protein levels of integrin αv and β3 in these cells (Figures S7A and S7B). These results suggest that integrins may be involved in RSPO2- induced FAK/SRC signal activation. In A2780 and OVCAR3 cells, presilencing of integrin αv/β3 abolished the enhancing effect of RSPO2 on FAK phosphorylation and partially diminished the activation of Src (Figures 6B, and S7C). Furthermore, knockdown of integrin αv/β3 also reduced the promotive effects of RSPO2 on cell adhesion and migration and partially blocked RSPO2-enhanced cancer cell growth (Figures 6C, S7D, and S7E). These results indicate that the upregulation of integrin αv/β3 participates in RSPO2-induced FAK/Src activation and tumor progression. Treatment with MG132 blocked the reduction of integrin αv/β3 in RSPO2-silenced ovarian cancer cells (Figure S7F). Moreover, higher levels of ubiquitinated integrin αv and β3 were observed in RSPO2-silenced cells than in control cells (Figure 6D). These results suggest that RSPO2 prevents the ubiquitination and degradation of integrin αv/β3. RSPOs have been shown to bind to several different intracellular signal proteins to regulate their stability (Carmon et al., 2011; Nam et al., 2006). Indeed, reciprocal interactions between RSPO2 and integrin αv and β3 were verified in both cell lines by Co-IP (Figures 6E and S7G). These results suggest that RSPO2 may serve as a binding ligand for integrin αv and/or β3. To show a direct physical interaction, we performed an *in vitro* pulldown assay using recombinant RSPO2 protein and found that purified RSPO2 pulled down integrin β3 but not integrin αv in cell lysates (Figure 6F). To further determine the specific regions of RSPO2 responsible for mediating its interaction with integrin β3, we generated mutants with two FUs or a C-terminal TSP domain. Co-IP assays demonstrated that both full-length RSPO2 and its FUs fragment bound specifically to LGR4 and integrin β3, whereas no binding of the TSP fragment to either LGR4 or integrin β3 was detected (Figure 6G). Furthermore, the FUs fragment potentiated the phosphorylation of both Src and FAK (Figure S7H). These results suggest that RSPO2 directly binds to integrin β3 via the FUs domain. Taken together, the above results support the hypothesis that RSPO2 acts as a ligand for integrin β3 and thus increases the stability of integrins, which induces downstream FAK/Src signaling activation and ovarian cancer progression.

## Discussion

**Figure 7:**
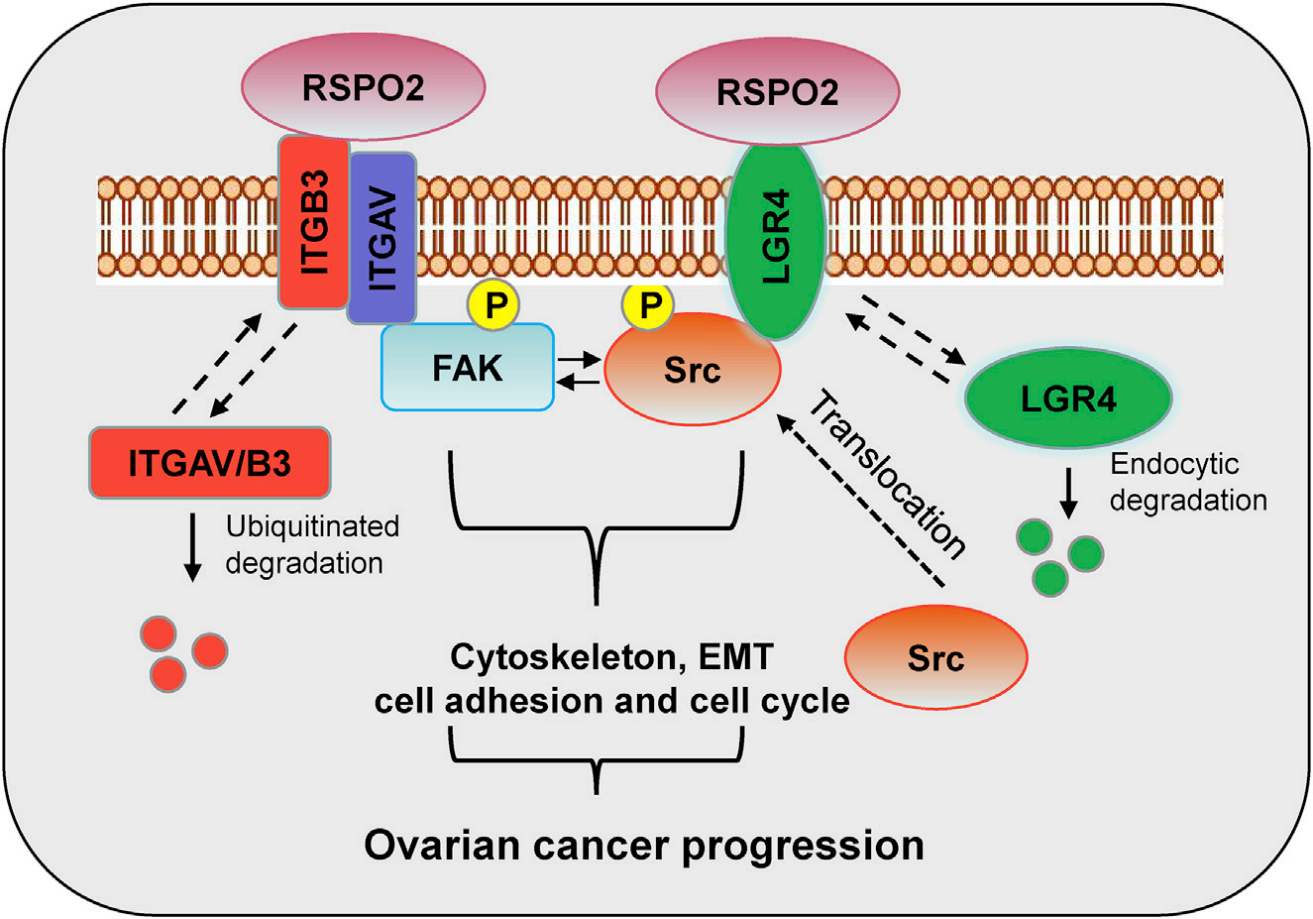
Schematic diagram illustrating the dual mechanism of RSPO2-mediated FAK/Src signaling activation In the presence of RSPO2, endocytic degradation of LGR4 is attenuated, which increases the accumulation of LGR4 on the plasma membrane, leading to plasma membrane translocation and autophosphorylation of Src. RSPO2 can also increase the stability of integrins by directly binding to integrin β3, leading to enhanced FAK phosphorylation. Constitutive activation of FAK/Src signaling promotes the growth and metastasis of ovarian cancer.

Recently, the importance of RSPO2 in development and tumorigenicity has been increasingly recognized (Ter Steege and Bakker, 2021). However, little is known about the function of RSPO2 in ovarian cancer. In this study, we demonstrated that RSPO2 plays an oncogenic role in ovarian cancer progression by promoting the growth and metastasis of ovarian cancer cells. Consistent with its oncogenic function, the RSPO2 protein level was increased in human ovarian tumor specimens and associated with poor prognosis. Mechanistically, we revealed that RSPO2 promotes ovarian cancer progression by enhancing FAK/Src signaling cascades via two unexpected actions. First, RSPO2 promoted LGR4-mediated recruitment of Src to the plasma membrane. Second, RSPO2 is directly bound to integrin β3 as a ligand and thus increased the stability of integrins, and both actions potentiate the autoactivation of FAK and/or Src in ovarian cancer cells. Based on our results, we proposed a novel Wnt-independent mechanism underlying the promotive effect of RSPO2 on ovarian cancer progression (Figure 7).

RSPOs have been shown to potentiate the Wnt/β-catenin signaling pathway in a variety of cancers (Ter Steege and Bakker, 2021), but whether RSPOs crosstalk with other intracellular signaling pathways to regulate tumor progression remains poorly understood. In the current study, we provided evidence that independent of Wnt signaling, RSPO2 promotes ovarian cancer progression by potentiating FAK/Src signaling cascades. Both FAK and Src are nonreceptor tyrosine kinases that are implicated in nearly every step of cancer progression (Sulzmaier et al., 2014; Frame, 2002). FAK/Src overexpression and/or activation occur in most epithelial ovarian cancers and are significantly associated with poor patient survival (Sood et al., 2004; Wiener et al., 2003; Huang et al., 2013). Via FAK/Src inhibitor treatment and functional rescue experiments, we validated the critical role of FAK/Src signaling activation in RSPO2-promoted ovarian cancer progression. Our data suggested that RSPO2-mediated hyperactivation of Src/Akt participates in cell cycle progression, thus providing a growth advantage to ovarian cancer cells. Moreover, we demonstrated that the inhibition of FAK activity largely reduced the promotive effects of RSPO2 on tumor metastasis indicators such as EMT, MMPs secretion, cell adhesion, and migration. These results suggest that FAK signal activation may primarily contribute to the RSPO2-triggered ovarian cancer metastasis. Inhibition of FAK or Src also partially impaired RSPO2-promoted cancer cell proliferation and migration, respectively, indicating that FAK/Src mutual activation in RSPO2-overexpressing tumor cells may synergistically promote tumor progression. Several studies have reported that aberrant activation of FAK/Src is an important cause of chemotherapeutic resistance in ovarian cancers (Levy et al., 2019; George et al., 2005; Halder et al., 2005). Here, we demonstrated that the pharmacological inhibition of RSPO2-induced FAK/Src activation significantly impaired ovarian cancer cell growth and migration, suggesting that the RSPO2/FAK/Src axis is a druggable target. Given that targeting tumor-derived RSPO2 with monoclonal antibodies effectively retarded the growth of patient-derived xenograft (PDX) tumors, including ovarian tumor (Chartier et al., 2016; Storm et al., 2016), the antibody-based RSPO2 blockage may be a useful adjuvant treatment to improve the outcomes of ovarian cancer patients with high RSPO2 expression.

Abnormal activation of Src has been documented in ovarian cancer, but the regulatory mechanism of Src activation has not been fully clarified (Simpkins et al., 2018; George et al., 2005). In the current study, we identified RSPO2 as a novel positive regulator of Src autoactivation. Activation of Src is tightly regulated by phosphorylation/dephosphorylation processes, and dephosphorylation of the Tyr527 residue results in its activation by the autophosphorylation of Tyr416 (Roskoski, 2005). Translocation of inactive Src to the plasma membrane is an alternative way to trigger Src autophosphorylation (Roskoski, 2015; Sandilands and Frame, 2008). Here, we found that RSPO2 stimulation increased the distribution of Src on the plasma membrane but did not affect the total amount of Src or the phosphorylation of Src at Tyr527. Our results suggest that the membrane translocation of Src is the main cause of Src activation in RSPO2-treated ovarian cancer cells. We showed that RSPO2 increased the distribution of LGR4 on the plasma membrane and thereby prevented its endocytic degradation. More importantly, we demonstrated that the presilencing of LGR4 counteracted RSPO2-induced Src hyperactivation and cell proliferation. Our study suggests that LGR4 is a key mediator in RSPO2-induced Src autoactivation. The RSPO-LGR4 complex is well-established to bind with intracellular Wnt signaling proteins such as ZNRF3, RNF43, and IQGAP1 to form supercomplex (Carmon et al., 2011, 2014). We validated a physical interaction between endogenous LGR4 and Src. On the other hand, we showed that RSPO2 was intimately associated with the interaction of LGR4 with Src. Our data showed for the first time that there is a physical interaction between LGR4 and Src, indicating that RSPO2, LGR4, and Src can form a supercomplex on the plasma membrane. Taken together; our study provides new mechanistic insights into the activation of Src in ovarian cancer cells: the RSPO2-LGR4 interaction increases the distribution of LGR4 on the plasma membrane as well as its association with Src, which, in turn, enhances Src recruitment to the inner surface of the plasma membrane for autoactivation.

Integrins regulate multiple cellular functions crucial to the growth and metastasis of solid tumors (Mitra and Schlaepfer, 2006). In ovarian tumors, altered integrins expression or abnormal activation of integrins has been shown to contribute to the proliferation, invasion, and chemoresistance of ovarian cancer cells (Davidson et al., 2003; Dolinschek et al., 2021; Lengyel, 2010). In this study, we found that overexpression of RSPO2 increased the total amounts of integrin-αv/β3 in ovarian cancer cells by preventing their ubiquitination and degradation. Several studies have claimed that the FAK/Src dual-kinase complex is a key adapter in integrin-mediated signal transduction pathways for tumor growth and metastasis (Mitra and Schlaepfer, 2006; Seguin et al., 2015). Consistent with these findings, we demonstrated that the presilencing of integrin-αv/β3 not only blocked RSPO2-induced FAK/Src phosphorylation but also attenuated the RSPO2-promoted tumor cell proliferation and migration. Our results suggested that integrin-mediated FAK/Src signaling activation plays a critical role in RSPO2-promoted ovarian cancer progression. Integrins are multifunctional receptors that exist as heterodimers composed of α and β subunits and bind to various ligands (Mitra and Schlaepfer, 2006). We demonstrated that RSPO2 enhanced the stability of integrins by direct physical interaction with integrin β3. Our data suggest that the secreted protein RSPO2 acts as a new ligand for integrin β3 and that the binding of RSPO2 to integrin β3 may enhance the stability of the integrin-αv/β3 heterodimer and thus activates downstream FAK/Src signaling. In this study, we further mapped the integrin β3 binding site in RSPO2 to its FUs domain, similar to the domain that interacts with LGRs. Intriguingly, a recent study showed that the binding of the TSP domain of RSPO2 to the ALK3 receptor inhibited BMP signaling in acute myeloid leukemia (AML) cells (Sun et al., 2021). Taken together, these findings suggest that RSPO2 is a multipotent ligand that can bind to different plasma membrane receptors through its FUs or TSP domain to regulate different cell signaling cascades.

In summary, we demonstrated that RSPO2 acts as an oncogene in ovarian cancer progression. We elucidated a Wnt-independent mechanism by which RSPO2 promotes ovarian cancer cell growth and metastasis through the potentiation of FAK/Src signaling. RSPO2 enhances the autoactivation of FAK and Src by binding with the specific receptors integrin β3 and LGR4, respectively. Our study suggests that the disruption of RSPO2/FAK/Src signaling cascades may be a therapeutic strategy for aggressive ovarian cancer.

### Limitations of the study

There are three major limitations in this study that could be addressed in future research. First, this study would be strengthened by including more high-grade ovarian cancer (HGOC) cell lines to consolidate the major findings. Second, the association between RSPO2 expression and clinical pathologic features of individuals could not be analyzed owing to the unavailability of sufficient paired ovarian tumor samples, and exploration with a large sample size is needed to further support this finding. In addition, further investigations are required to elucidate the underlying mechanism of how RSPO2 expression is upregulated in ovarian cancer tissues.

## STAR★Methods

### Key resources table

**Table:** 

REAGENT or RESOURCE	SOURCE	IDENTIFIER
**Antibodies**		

Phospho-Akt (Ser473) Antibody	Cell Signaling Technology	Cat# 9271; RRID: AB_329825
Phospho-GSK3β(Ser9) (D85E12) XP Rabbit mAb	Cell Signaling Technology	Cat# 5558; RRID: AB_10013750
Phospho-p44/42 MAPK(Erk1/2)(Thr202/Tyr204) Antibody	Cell Signaling Technology	Cat# 9101; RRID: AB_331646
Phospho-SAPK/JNK(Thr183/Tyr185)(G9) Mouse mAb	Cell Signaling Technology	Cat# 9255; RRID: AB_2307321
SAPK/JNK Antibody	Cell Signaling Technology	Cat# 9252; RRID: AB_2250373
Phospho-Src(Tyr527) Antibody	Cell Signaling Technology	Cat# 2105; RRID: AB_331034
Phospho-Src Family(Tyr416) Antibody	Cell Signaling Technology	Cat# 2101; RRID: AB_331697
Akt(pan)(C67E7) Rabbit mAb	Cell Signaling Technology	Cat# 4691; RRID: AB_915783
Axin1(C76H11) Rabbit mAb	Cell Signaling Technology	Cat# 2087; RRID: AB_2274550
Axin2(76G6) Rabbit mAb	Cell Signaling Technology	Cat# 2151; RRID: AB_2062432
CyclinE1(D7T3U) Rabbit mAb	Cell Signaling Technology	Cat# 20808; RRID: AB_2783554
β-catenin Antibody (Carboxy-terminal Antigen)	Cell Signaling Technology	Cat# 9587; RRID: AB_10695312
CyclinD1(DCS-6)	Santa Cruz Biotechnology	Cat# sc-20044; RRID: AB_627346
Anti-GPCR GPR48 antibody(LGR4)	Abcam	Cat# ab75501; RRID: AB_1523714
c-Myc(9E10)	Santa Cruz Biotechnology	Cat# sc-40; RRID: AB_2892598
p44/42 MAPK(Erk1/2) Antibody	Cell Signaling Technology	Cat# 9102; RRID: AB_330744
PTEN(138G6) Rabbit mAb	Cell Signaling Technology	Cat# 9559; RRID: AB_390810
R-Spondin2(C-12)	Santa Cruz Biotechnology	Cat# sc-74883; RRID: AB_1568287
Src(36D10) Rabbit mAb	Cell Signaling Technology	Cat# 2109; RRID: AB_2106059
Purified Mouse Anti-E-Cadherin	BD Biosciences	Cat# 610181; RRID: AB_397580
Purified Mouse Anti-N-Cadherin	BD Biosciences	Cat# 610920; RRID: AB_2077527
GAPDH(14C10) Rabbit mAb	Cell Signaling Technology	Cat# 2118; RRID: AB_561053
p-EGFR(Tyr1068) (D7A5)	Cell Signaling Technology	Cat# 3777; RRID: AB_2096270
EGFR	Cell Signaling Technology	Cat# 2232; RRID: AB_331707
Z0-1(H-300)	Santa Cruz Biotechnology	Cat# sc-10804; RRID: AB_2205514
p-FAK(Tyr397)	Cell Signaling Technology	Cat# 3283; RRID: AB_2173659
β-actin	Cell Signaling Technology	Cat# 4970; RRID: AB_2223172
FAK	Cell Signaling Technology	Cat# 71433; RRID: AB_2799801
MMP7	Abcam	Cat# ab39984; RRID: AB_776498
MMP2	Abcam	Cat# ab80737; RRID: AB_1603130
LGR5	ABGENT	Cat# AP2745d; RRID: AB_2281170
Integrin αv	Santa Cruz Biotechnology	Cat# sc-9969; RRID: AB_627116
Integrin α4	Santa Cruz Biotechnology	Cat# sc-365209; RRID: AB_10709586
Integrin α5	Santa Cruz Biotechnology	Cat# sc-376199; RRID: AB_10987904
Integrin β1	Santa Cruz Biotechnology	Cat# sc-374429; RRID: AB_11012020
Integrin β3	Santa Cruz Biotechnology	Cat# sc-365679; RRID: AB_10844835
HA Epitope Tag Antibody	Novus Biologicals	Cat# NB600-363; RRID: AB_10001504
Myc-tag Rabbit mAb	Cell Signaling Technology	Cat# 2278; RRID: AB_490778
Myc-tag Mouse mAb	Cell Signaling Technology	Cat# 2276; RRID: AB_331783
DYKDDDDK Tag (FLAG)	Cell Signaling Technology	Cat# 8146; RRID: AB_10950495
His-tag	Cell Signaling Technology	Cat# 12698; RRID: AB_2744546
Fibronectin	BD transduction laboratories	Cat# 610077; RRID: AB_2105706
Normal Rabbit IgG	Cell Signaling Technology	Cat# 2729; RRID: AB_1031062
Mouse mAb IgG1 Isotype Control	Cell Signaling Technology	Cat# 5415; RRID: AB_10829607
LAMP1	Santa Cruz Biotechnology	Cat# sc-20011; RRID: AB_626853
Frizzled6 (D16E5) Rabbit mAb	Cell Signaling Technology	Cat# 5158; RRID: AB_10621242
Anti-Frizzled 7 antibody	Abcam	Cat# ab64636; RRID: AB_1640522

**Biological samples**		

Human ovarian cancer and paired nontumor ovarian tissues	Shanghai Superbiotek Inc. (China)	http://www.superbiotek.com

**Chemicals, peptides, and recombinant proteins**		

Recombinant human RSPO2 protein	R&D Systems (USA)	3266-rs-025/cf
Wnt3a	R&D Systems (USA)	5036-WN-500
LY294002	Selleck	S1105
Cycloheximide (CHX)	Sigma	C7698
Defactinib	RayStar Biosystems (China)	1073154-85-4
Saracatinib	Selleck	S1006
Niclosamide	Sigma-Aldrich	50-65-7
MTT	Sangon Biotech	TB0799-1G-N
vitronectin	Sigma-Aldrich	5051
MG132	Sigma-Aldrich	M7449

**Critical commercial assays**		

Dual-Luciferase Reporter Assay System	Promega	Cat# E1910
Pierce Cobalt kit	Thermo Fisher Scientific	Cat# #21277

**Deposited data**		

The RNA-seq data for A2780 cells upon RSPO2 overexpression	This paper	Accession number: PRJNA783149 BioSample accessions: SAMN24619039, SAMN24619040 locus tag prefix: LQK87 (SAMN24619040) https://www.ncbi.nlm.nih.gov/sra/PRJNA783149

**Experimental models: Cell lines**		

Human: A2780	ATCC	CVCL_0134
Human: OVCAR3	ATCC	CVCL_0465
Human: HEK293T	ATCC	CVCL_0063

**Experimental models: Organisms/strains**		

BALB/c-nude mice	Vital River Experimental Animal Center (Beijing, China)	N/A

**Oligonucleotides**		

siRNA targeting sequence for RSPO2, LGR4, ITGAV, and ITGB3, see Table S1	This paper	N/A
shRNA targeting sequence for RSPO2, see Table S1	This paper	N/A
Primers for FUs and TSP, see Table S3	This paper	N/A
Primers for qRT-PCR, see Table S3	This paper	N/A

**Recombinant DNA**		

Plasmid: RSPO2	Wu et al. (2014)	N/A
Plasmid: FUs	This paper	N/A
Plasmid: TSP	This paper	N/A
Plasmid: LGR4	Qiang Hou (Wenzhou Medical University, China)	N/A
Plamid: ITGAV	Miaoling Biotech (China)	Cat# P40612
Plamid: ITGB3	Miaoling Biotech (China)	Cat# P39193

**Software and algorithms**		

GraphPad Prism Software	GraphPad	https://www.graphpad.com
R2: Genomics Analysis and Visualization Platform	R2	http://r2.amc.nl, http://r2platform.com

### Resource availability

#### Lead contact

Information and requests for resources should be directed to and will be fulfilled by the lead contact, Xincheng Lu (xinchenglu@yahoo.com).

#### Materials availability

This study did not generate new unique reagents.

### Experimental model and subject details

#### Cell culture

The A2780, OVCAR3 and HEK293T cell lines were originally obtained from the American Type Culture Collection (ATCC, USA). All cells were cultured in the recommended medium (DMEM for A2780 and RPMI-1640 for HEK293T and OVCAR3 cells) supplemented with 10% fetal bovine serum (FBS) and 1% penicillin/streptomycin at 37°C in 5% CO_2_. The A2780, OVCAR3 and HEK293T cell lines had recently been authenticated using short tandem repeat DNA profiling, and all cell lines tested negative for mycoplasma contamination before the experiment.

#### Reagents and antibodies

Recombinant human RSPO2 protein (3266-rs-025/cf) and Wnt3a (5036-WN-500) was purchased from R&D Systems (USA). The FAK inhibitor defactinib (1073154-85-4) was purchased from RayStar Biosystems (China). The Src inhibitor saracatinib (S1006) and Akt inhibitor LY294002 (S1105) were purchased from Selleck (China). The Frizzled inhibitor Niclosamide was purchased from Sigma-Aldrich (St. Louis, MO). Cycloheximide(CHX) was purchased from Sigma (C7698). SiRNAs targeting the open reading frames of RSPO2, LGR4, ITGAV, and ITGB3 were synthesized by GenePharma (Shanghai, China). Lentivirus containing shRNA against RSPO2 was designed and produced by GeneChem Co. (Shanghai, China), siRNA and shRNA sequences are listed in Table S1. Cells grown to a confluence of 50–70% were transfected with siRNA using Lipofectamine RNAiMAX (Invitrogen) according to the manufacturer’s instructions and plated again for further experiments. The knockdown efficiency was determined using qRT-PCR or western blotting. *All antibodies were purchased from commercial manufacturers, and detailed information is listed in* Table S2.

#### Plasmid constructs

The construction of the myc-tagged full-length RSPO2 expression plasmid was described previously (Wu et al., 2014). To construct RSPO2 mutants, the following amino acid sequences of RSPO2 were subcloned into the pcDNA3.1 vector: FUs, 1–146; TSP, 145–243. The TOPFlash and pRL-TK plasmids were obtained from Promega (E2241). The flag-tagged ITGAV (NM_002210.5:284–3430) and ITGB3 (NM_000212.2) expression plasmid was purchased from Miaoling Biotech (China). The myc-tagged LGR4 expression plasmid was a gift from Qiang Hou (Wenzhou Medical University, China). The primer sequences used for plasmid construction are listed in Table S3.

#### Mice

Female athymic nude mice were purchased from Vital River Experimental Animal Center (Beijing, China) and housed under pathogen-free conditions. All *in vivo* experiments and protocols were approved by the Institutional Animal Care and Use Committee of Wenzhou Medical University.

#### Patient samples

Ovarian tissue samples or microarrays containing human ovarian cancer and paired nontumor ovarian tissues were purchased from Shanghai Superbiotek Inc. (China), with appropriate Institutional Review Board approval and informed patient consent.

### Method details

#### MTT and clonogenic assays

To obtain stable RSPO2 transfectants, transfected cells were cultured in G418-containing medium, resistant clones were pooled, and the expression of RSPO2 was confirmed by quantitative reverse transcription–PCR (qRT-PCR) and Western blotting. For the 3-(4,5-dimethylthiazol-2-yl)-2,5-diphenyltetrazolium bromide (MTT) assay, cells were incubated with MTT (Sangon Biotech, TB0799-1G-N) solution (final concentration, 5 mg/mL) for 5 h, and cell viability was analyzed as described previously (Wu et al., 2014). For the clonogenic assay, 1 × 10 ^3^ cells were seeded in ṣix-well plates in triplicate. At the end of the experiment, colonies (≥50 cells) were counted after staining with 0.5% crystal violet in 20% methanol.

#### Transwell migration and invasion assays

The Transwell migration assay was performed using Corning chambers (Corning, 3422). A total of 1 × 105 cells in 100 μL of serum-free medium were seeded in the upper compartment of the chamber, whereas 600 μL of complete medium containing 10% FBS was placed in the lower compartment of the chamber. After incubation for 24 h, the cells that migrated to the bottom surface of the membrane were fixed with methanol and stained with 0.5% crystal violet. Cells in five randomly selected fields of the membrane were counted under an inverted microscope. The Transwell invasion assay was performed following the same protocol used for the migration assay except that Corning Matrigel Invasion Chambers (Corning, 354480) were used.

#### Wound healing and cell adhesion assays

The protocol for the wound healing assay was described previously (Dong et al., 2017). For the cell adhesion assay, 96-well plates were precoated with 50 μg/mL vitronectin (Sigma, 5051) overnight at 4°C. Cells were resuspended in serum-free medium, and 50,000 cells were seeded in the coated plates for 2 h at 37°C. After washing twice with PBS, adherent cells were stained with 0.5% crystal violet in 20% methanol, photographed, and solubilized in 100% ethanol. The absorbance at 540 nm was measured in a microplate reader.

#### Xenograft model

Female athymic nude mice were purchased from Vital River Experimental Animal Center (Beijing, China) and housed under pathogen-free conditions. All *in vivo* experiments and protocols were approved by the Institutional Animal Care and Use Committee of Wenzhou Medical University. A2780 cells (3 × 10^6^ cells suspended in 200 μL of PBS) infected with lentivirus were injected subcutaneously into nude mice Tumor volume was measured every two days with a caliper and calculated using the standard equation: V = A × B^2^ × 0.5326 (A = long axis and B = short axis). RSPO2-overexpressing or control A2780 or OVCAR3 cells were implanted orthotopically (1 × 10^6^ cells suspended in 20 μL of PBS) into the left ovaries of mice for 5 weeks. At the end of the experiments, all mice were euthanized, and subcutaneous or metastatic tumors were harvested and photographed. The tumor weight, ascites weight, and number of nodules were recorded. Tumor tissues were then fixed in formalin for paraffin embedding or were snap frozen.

#### Western blot analysis

Protein preparation and concentration determination were performed as described previously (Dong et al., 2017). Nuclear proteins were isolated using NE-PER™ Nuclear and Cytoplasmic Extraction Reagents (Pierce, 78833) according to the manufacturer’s instructions. Proteins were separated using sodium dodecyl sulfate–polyacrylamide gel electrophoresis (SDS-PAGE) and transferred onto polyvinylidene difluoride membranes (Bio-Rad, 1620177). After blocking with 5% milk in TBS containing 0.1% Tween 20 (TBST), the membranes were incubated with the corresponding primary antibodies (dilutions are listed in Table S2) followed by horseradish peroxidase (HRP)-conjugated secondary antibodies. Protein bands were visualized with an Immun-Star HRP Chemiluminescence Kit (Bio-Rad, 1705061).

#### Immunohistochemistry (IHC)

Immunohistochemical staining of tissue microarrays was performed with an antibody against RSPO2 (1:200). The stained tissue microarray chips were digitally scanned, and the levels of RSPO2 were scored semiquantitatively. The immunohistochemical score was determined by multiplying the intensity score by the positive staining score of the cells. The intensity score was assigned as follows: 0 = negative staining, 1 = weak staining, 2 = moderate staining, or 3 = strong staining. The positive staining score was defined as the percentage of cells positive for RSPO2 staining. Paraffin-embedded tumor nodules were sectioned and stained with primary antibody against RSPO2 (1:200) followed by a biotinylated and peroxidase-conjugated secondary antibody. The stained slides or tissue microarray chips were visualized and imaged with a Zeiss microscope with a 10× or 40× objective lens.

#### Immunofluorescence imaging

Immunofluorescence analysis was performed as described previously (Wu et al., 2014). In brief, cells were seeded on coverslips for 48 h and treated with or without exogenous RSPO2 protein. After gentle washes with PBS, cells were fixed with 4% formaldehyde and permeabilized with 0.5% Triton X-100 in PBS. Cells were subsequently blocked with 2% bovine serum albumin in PBS containing 0.1% Triton X-100 prior to incubation with antibodies against the Myc tag (Cell Signaling Technology, #2278), lysosomal-associated membrane protein-1 (LAMP1; Santa Cruz Biotechnology, sc-20011), integrin β3 (Santa Cruz Biotechnology, sc-365679), integrin αv (Santa Cruz Biotechnology, sc-9969), β-catenin (Cell Signaling Technology, #9587) (diluted 1:100) and Src (Cell Signaling Technology, #2101) (diluted 1:400) at 4°C overnight. After three washes with PBST, samples were incubated with Alexa Fluor 488- or 594-conjugated secondary antibodies (diluted 1:500). Filamentous actin (F-actin) was stained with Acti-stain 488 phalloidin (diluted 1:50, Thermo Fisher Scientific, A12379). Coverslips were mounted on glass slides in the presence of DAPI for nuclear staining, and cell images were acquired with a confocal microscope via Nikon NIS-Elements software.

#### Coimmunoprecipitation (Co-IP)

Cells were washed with ice-cold PBS and lysed in immunoprecipitation assay buffer (50 mM Tris-HCl (pH 7.5), 150 mM NaCl, 1% Triton X-100, 1 mM EDTA, and 10% glycerol) supplemented with Protease/Phosphatase Inhibitor Cocktail (Cell Signaling Technology, #5872). Lysates were incubated on ice for 20 min and centrifuged at 12,000 rpm for 20 min. Cell lysates were incubated first with the corresponding primary antibody overnight at 4°C and then with Protein G-Sepharose (GE Healthcare, 17-0618-01) for 3 h at 4°C. The beads were washed four times with immunoprecipitation assay buffer, suspended in Laemmli buffer, and boiled for 5 min. Samples were analyzed by Western blotting with the indicated antibodies.

#### *In vitro* pulldown assays

His-tagged recombinant human RSPO2 protein (R&D System, 3266-rs-025/CF) and cell lysates from HEK293T cells overexpressing Flag-tagged Integrin αv or Integrin β3 were used for His-pulldown assays according to the instructions from a Pierce Cobalt kit (Thermo Fisher Scientific, #21277). His-tagged RSPO2 (approximately 20 μg of protein) was bound to Ni^2+^-NTA agarose beads. After washing with wash buffer, the agarose beads were incubated for 3 h at 4°C with 200 μg of HEK293T cell extract containing the potential interacting Flag-tagged fusion protein. After washing again, bound proteins were analyzed by Western blotting. Anti-His (Cell Signaling Technology, #12698) and anti-Flag (Cell Signaling Technology, #8146) primary antibodies were used.

#### Ubiquitination assay

The ubiquitination assay was performed as described previously (Dong et al., 2017). In brief, cells were transfected with the HA-Ubiquitin plasmid. Twenty-four hours after transfection, cells were treated with the proteasome inhibitor MG132 (25 mM) (Sigma, M7449) for 4 h and were then lysed in ubiquitination assay buffer containing protease/phosphatase inhibitors. Cell lysates were clarified and incubated with an anti-integrin αv or β3 antibody overnight at 4°C. Immunocomplexes were incubated with Protein G-Sepharose (GE Healthcare) for another 3 h at 4°C, washed four times with wash buffer, and boiled for 5 min in Laemmli buffer before separation by SDS-PAGE. Western blotting was performed with an anti-HA antibody to detect ubiquitinated integrin αv or β3.

#### RNA extraction and qRT-PCR

Cells were lysed, total RNA was purified using TRIzol Reagent (Invitrogen, 15596018), and reverse transcription was performed using an M-MLV reverse transcriptase kit (Invitrogen, 28025-013). qRT-PCR was carried out with SYBR Green (Tiangen, China, FP202-02) in biological triplicate in an ABI 7500 Real-Time detection system (Applied Biosystems) according to the manufacturer’s protocol. The primer sequences are listed in Table S1. Relative quantification of mRNA expression was performed using the comparative threshold cycle (C_t_) method with normalization to GAPDH. When necessary, we converted ▵▵C_t_ values to expression fold change values using the formula 2^-▵▵Ct^.

#### Cell cycle analysis by FACS

The cell cycle was analyzed by flow cytometry. In brief, a total of 5 × 10^5^ cells were seeded in 6-well plates and cultured for 24 h. The adherent cells were collected, washed twice with PBS, fixed with ice-cold 70% ethanol, and stored at −20°C overnight. Cell pellets were centrifuged at 1000 rpm for 5 min and were then washed with cold PBS; suspended in 500 mL of PBS containing 50 mg/mL propidium iodide, 0.1 mg/mL RNase A and 0.05% Triton X-100; and incubated at 37°C for 40 min in the dark. The cell cycle distribution was analyzed in a Becton Dickinson FACSCalibur flow cytometer. The experiment was repeated three times under the same conditions.

#### RNA sequencing (RNA-seq)

Total RNA was isolated from vector control A2780 cells and A2780 cells stably overexpressing RSPO2 using TRIzol reagent (Invitrogen). RNA was reverse transcribed to cDNA, and cDNA was amplified and fragmented. The final products were sequenced on the Illumina HiSeq 4000 or X Ten platform (BGI-Shenzhen, China). The RNA-seq data are available under NCBI Bioproject ID: PRJNA783149. DESeq2 (http://www.bioconductor.org/packages/release/bioc/html/DESeq2.htm) was used to perform differential expression analysis.

Phyper (https://en.wikipedia.org/wiki/Hypergeometric_distribution) was used to perform KEGG enrichment analysis of annotated differentially expressed genes.

#### TOPFlash reporter assay

Cells were transfected with TOPFlash and Renilla-TK (Promega, E2241) plasmids using Lipofectamine 2000 reagent (Invitrogen, 11668019). Forty-eight hours after transfection, the luciferase reporter assay was performed using a Dual Luciferase Reporter Assay System (Promega, E1910). Luminescence data are presented as the firefly luminescence intensity normalized to the Renilla luminescence intensity and then to the control condition.

### Quantification and statistical analysis

Data are presented as the mean ± SD values. Statistical analysis was performed using GraphPad Prism 8.0 and SPSS Statistics software (SPSS 20). Kaplan–Meier survival plots were generated using R2: Genomics Analysis and Visualization Platform (http://r2.amc.nl, http://r2platform.com). Kaplan Scan (KaplanScan) was used to find the most significant expression cutoff for survival analysis based on statistical testing. The Kaplan scanner separates the samples of tumor ovarian serous cystadenocarcinoma from TCGA dataset or GSE26193 dataset into two groups (high/low) based on the mRNA expression of RSPO2. Two-tailed Student’s *t* test was used for comparisons between different groups. Values of p < 0.05 were considered statistically significant. Error bars represent standard error from at least three biological replicates.

## Data Availability

• Data: All data generated or analyzed during this study are included in this published article. All relevant data are available from the lead contact upon request. • **Code**: This paper does not report original code. • **RNAsequencing data:** The RNA-sequencing data for A2780 cells upon RSPO2 overexpression has been deposited in NCBI Bioproject database under the accession number PRJNA783149, BioSample accessions: SAMN24619039, SAMN24619040, locus tag prefix: LQK87 (SAMN24619040), which can be accesses using the following link: https://www.ncbi.nlm.nih.gov/sra/PRJNA783149.
