# Supplementary material for: RSPO2 promotes progression of ovarian cancer through dual receptor-mediated FAK/Src signaling activation
Source: iScience. 2022 Sep 23;25(10):105184. doi: 10.1016/j.isci.2022.105184 (PMC9547309; doi:10.1016/j.isci.2022.105184)

iScience, Volume *25*

Supplemental information

RSPO2 promotes progression of ovarian cancer through dual receptor-mediated FAK/Src signaling activation

Rulu Pan, Yan Yu, Haiyan Zhu, Wenyi Zhang, Yuan Qin, Lin Ye, Juji Dai, Ren Huang, Xinyan Peng, Siqi Ye, Ziqi Lin, Shishun Huang, Shuyi Chong, Liting Lu, and Xincheng Lu

Supplemental Materials

Title:

RSPO2 promotes progression of ovarian cancer through dual receptor-mediated FAK/Src signaling activation

Pan R. et al.

Supplemental figure legends

Figure S1. Effects of RSPO2 knockdown or overexpression on ovarian cancer cell proliferation (Related to Figure 2).

(A) Stable ectopic overexpression of RSPO2 was verified by qRT-PCR (left) and western blot analysis (right) in A2780 and OVCAR3 cells stably overexpressing RSPO2 (RS2, pooled) or empty vector (Vec). (B) An MTT assay was conducted to evaluate the effect of stable overexpression of RSPO2 on cell proliferation. (C) qRT-PCR analysis of the knockdown efficiency of RSPO2 siRNA. A2780 and OVCAR3 cells were transfected with a scrambled nontargeting sequence (siNC) or siRNAs against RSPO2 (siRSPO2-1#, siRSPO2-2#). (D) The efficiency of lentivirus-mediated RSPO2 knockdown was validated by qRT-PCR (left) and Western blot analysis (right). ShRSPO2 was designed using the most effective interference sequence, siRSPO2-2# (shRS2). (E) The effect of siRNA-mediated RSPO2 knockdown on cell proliferation was determined by an MTT assay. (F) The effect of lentivirus-mediated RSPO2 knockdown on cell proliferation was determined by an MTT assay. *p < 0.05, **p < 0.01, p*** < 0.001 vs. control; two-tailed Student's

t-test. Error bars indicate mean ± SD.

Figure S2. Knockdown of RSPO2 suppresses migration and invasion of ovarian cancer cells in vitro and tumorigenesis in vivo (Related to Figure 2).

(A-B) Lentivirus-mediated RSPO2 knockdown suppresses the migration and invasion of ovarian cancer cells. Representative images (upper panel) and quantification (lower panel) of the Transwell assay results were presented. (C-D) RSPO2 knockdown suppressed tumor growth in nude mice (n = 5 per group). A2780 cells stably infected with lentivirus containing shRS2 or nontargeting shNC were inoculated into the dorsal flanks of nude mice (n = 5 mice per group). C, Tumor volumes were measured on the indicated days. D, Tumors were harvested, photographed, and weighed 30 days post inoculation. (E) Representative images showing immunohistochemical staining for the RSPO2 protein in disseminated tumor nodules in Fig. 2F. Scale bar, 50 μm. *p < 0.05 and **p < 0.01 vs. shNC; two-tailed Student's t-test. Error bars indicate mean ± SD.

Figure S3. RSPO2 does not potentiate Wnt signaling in ovarian cancer cells (Related to Figure 4).

(A) TOPFlash assay of A2780 and OVCAR3 cells stably overexpressing RSPO2 (RS2, pooled) or empty vector (Vec). (B) Wnt3a and RSPO2-induced β-catenin activity as measured by the TOPFlash assay in A2780, OVCAR3 and HEK293T cells. Cells were treated with 50 ng/mL Wnt3a and/or 200 ng/mL RSPO2 for 6 hr. (C) The effect

of RSPO2 overexpression on the expression of Wnt signaling proteins. (D) RSPO2 overexpression failed to promote nuclear translocation of β-catenin. β-catenin was detected using immunofluorescence staining and confocal microscopy. Scale bar, 20 μm. (E) The effects of RSPO2 overexpression on the expression of Frizzled 6/7 proteins. (F) The effects of siRNA-mediated RSPO2 knockdown on the expression of Frizzled 6/7 proteins. (G-H) Effects of Frizzled inhibitor Niclosamide (Niclo) on cell proliferation (G) and migration (H). A2780 and OVCAR3 cells stably overexpressing RSPO2 (RS2, pooled) or empty vector (Vec) were treated with DMSO or Niclosamide (1.0 μM) for 24 hr. *p < 0.05, **p < 0.01, p*** < 0.001 vs. control; two-tailed Student's t-test. Error bars indicate mean ± SD.

Figure S4. RSPO2 potentiates activation of FAK/Src/Akt in ovarian cancer cells (Related to Figure 4).

(A) Effect of RSPO2 overexpression on MAPK signaling pathway activation. The phosphorylation levels of ERK (Thr202/Tyr204) and JNK (Thr183/Tyr185) were analyzed by Western blotting. (B) Stimulation with RSPO2 protein potentiated the phosphorylation of FAK, Src, EGFR, and Akt. Cells were treated with the indicated concentrations of recombinant RSPO2 protein for 12 hr. (C) The phosphorylation of FAK, Src and Akt in ovarian tumor nodules from the orthotopic mouse model was analyzed by Western blotting. 1-2-3 means metastatic tumor nodules from different mice. (D) Src inhibitor saracatinib treatment attenuated the RSPO2-induced migration. Cells were treated with DMSO or saracatinib (Sar, 10 μM) for 1 hr. The

Representative images (upper panel) and quantification (lower panel) of the Transwell assay results were presented. (E) Effect of FAK inhibitor defactinib on RSPO2-induced cell proliferation. Cells were treated with DMSO or 1.0 μM defactinib (Def) for 3 hr. The representative images (upper panel) and quantification (lower panel) of the colony formation assay results were presented. (F) Representative images (upper panel) and quantification (lower panel) of colony formation assay results showing that the Akt inhibitor LY294002 abolished the promotive effect of RSPO2 overexpression on cell proliferation. A2780 and OVCAR3 cells stably overexpressing RSPO2 (RS2, pooled) or empty vector (Vec) were treated with DMSO or LY294002 (20 μM) for 24 hr. (G) LY294002 treatment abolished the RSPO2-induced increases in Cyclin D1 and E1 expression. Cells were treated with DMSO or LY294002 (20 μM) for 24 hr. *p < 0.05, **p < 0.01, p*** < 0.001 vs. control; two-tailed Student's t-test. Error bars indicate mean ± SD.

Figure S5. RSPO2 affects the expression and cellular distribution of LGR4 in ovarian cancer cells (Related to Figure 5).

(A) The protein levels of LGR4 and LGR5 in A2780 and OVCAR3 cells following RSPO2 protein treatment were analyzed by Western blotting. Cells were treated with the indicated concentrations of recombinant RSPO2 protein for 12 hr. (B) LGR4 expression in ovarian tumor nodules from the orthotopic mouse model was analyzed by Western blotting. 1-2-3 means metastatic tumor nodules from different mice. (C) Western blotting analysis of the knockdown efficiency of LGR4 in A2780 and

OVCAR3 cells. Cells were transfected with siRNA against LGR4 (siLGR4) or a scrambled nontargeting sequence (siNC). (D) The effect of LGR4 knockdown on RSPO2-induced cell migration. The representative images (upper panel) and quantification (lower panel) of the Transwell assay results were presented. (E) qRT-PCR analysis of LGR4 mRNA levels in A2780 and OVCAR3 cells stably overexpressing RSPO2 (RS2, pooled) or vector (Vec). (F) Fluorescence microscopy analysis of the cellular localization of LGR4 following RSPO2 protein treatment. LAMP1 was used as a lysosome marker, and cells were treated with PBS (vehicle) or recombinant RSPO2 protein (200 ng/ml) for 2 hr. Scale bar, 10 μm. *p < 0.05, **p < 0.01, p*** < 0.001 vs. control; two-tailed Student’s t-test. Error bars indicate mean ± SD.

Figure S6. LGR4 participates in the RSPO2-potentiated Src activation (Related to Figure 5).

(A) The effects of LGR4 overexpression on the phosphorylation levels of Src and Akt were analyzed by Western blotting. A2780 and OVCAR3 cells were transiently transfected with empty vector (Vec) or the LGR4 expression plasmid for 48 hr. (B) Coexpression of RSPO2 and LGR4 sensitized cells to RSPO2- or LGR4-induced Src activation. Vector (-), RSPO2 and LGR4 plasmids were transiently transfected into cells alone or in combination for 48 hr. (C) Co-IP analysis of the interactions of RSPO2 with LGR4 and Src. A2780 and OVCAR3 cells were transfected with Myc-tagged RSPO2, and cell lysates were then immunoprecipitated with an anti-Myc

(RSPO2) antibody and probed with anti-Myc, anti-LGR4 or anti-Src antibodies.

Figure S7. Integrin αv/β3 participates in RSPO2-mediated ovarian cancer progression (Related to Figure 6).

(A) Western blot analysis of integrin αv and β3 expression in ovarian cancer cells stably overexpressing RSPO2 (RS2, pooled) or vector control (Vec). (B) The expression levels of integrin αv and β3 were detected by immunofluorescence imaging. Scale bar, 10 μm. (C) The knockdown efficiency of the integrin αv and β3 siRNAs was verified by Western blot analysis. Cells were transfected with siRNA against integrin αv, β3, or both (siITGAV, siITGB3 or siITGAV/B3, respectively) or a scrambled nontargeting sequence (siNC). (D) Knockdown of integrin αv/β3 diminished the promotive effect of RSPO2 on cell adhesion. Adhesion and cell attachment, as evaluated by cellular binding to vitronectin, were quantitatively analyzed in cells with stable expression of control vector (Vec) or RSPO2 (RS2) and depletion of integrin αv/β3 (siITGAV/B3). The data are presented as the mean ± SD of a representative experiment performed in triplicate. ***$p < 0.001$ vs. control (Vec); two-tailed Student's t-test. Error bars indicate mean ± SD. (E) The effect of ITGAV/B3 knockdown on RSPO2-induced cell proliferation. The representative images (upper panel) and quantification (lower panel) of the colony formation assay results were presented. (F) MG132 treatment blocked the downregulation of integrin αv and β3 following RSPO2 interference. OVCAR3 cells were pretreated with MG132 (10 μM) for 4 hr and were then transfected with siRNA against RSPO2

(siRS2) or siNC for 48 hr. (G) Co-IP analysis of the interactions between ectopic RSPO2 (Myc-tagged) and endogenous integrin αv and β3 in A2780 and OVCAR3 cells. (H) Potentiating effects of RSPO2 mutants on the phosphorylation of FAK and Src. Cells were transiently transfected with empty vector (Vec), Myc-tagged RSPO2 (WT) or Myc-tagged mutants containing two furin domains (FUs) or a TSP-1 domain (TSP) for 48 hr. *p < 0.05, **p < 0.01, p *** <0.001; two-tailed Student's t-test. Error bars indicate mean ± SD.

Table S1. The targeting sequences used for siRNA and shRNA interference (Related to Figure 2-6).

.

Target gene	Sequence (5'-3')	Application
RSPO2-1#	AGACGCAGUAAGCGAGCUA	Gene silence
RSPO2-2# (shRSPO2)	GUUGGUCAUUGGAGCGAAUTT	Gene silence
LGR4-1#	AAGUAAACUGUGGUCAAUUTT	Gene silence
LGR4-2#	GUAGAAACCUGAUACAUGATT	Gene silence
ITGAV-1#	GAGGUCGAAACAGGAUAAATT	Gene silence
ITGAV-2#	GUCGUCUGCUUCAUUUAAUTT	Gene silence
ITGB3-1#	CCGCUUCAAUGAGGAAGUGAA	Gene silence
ITGB3-2#	CAAGCUGAACCUAAUAGCCAU	Gene silence
Nontarget control siRNA	UGGUUUACAUGUCGACUAA	Gene silence

Table S2. Primary antibodies used (Related to Figure 1-6).

Antibody	LOT	kD	Host	Company	Application
Phospho-Akt (Ser473) Antibody	#9271	60	Rabbit	CST	WB 1:2000
Phospho-GSK3β(Ser9) (D85E12) XP Rabbit mAb	#5558	46	Rabbit	CST	WB 1:2000
Phospho-p44/42 MAPK(Erk1/2)(Thr202/Tyr204) Antibody	#9101	42,44	Rabbit	CST	WB 1:2000
Phospho-SAPK/JNK(Thr183/Tyr185)(G9) Mouse mAb	#9255	46,54	Mouse	CST	WB 1:2000
SAPK/JNK Antibody	#9252	46,54	Rabbit	CST	WB 1:2000
Phospho-Src(Tyr527) Antibody	#2105	60	Rabbit	CST	WB 1:2000
Phospho-Src Family(Tyr416) Antibody	#2101	60	Rabbit	CST	WB 1:2000
Akt(pan)(C67E7) Rabbit mAb	#4691	60	Rabbit	CST	WB 1:2000
Axin1(C76H11) Rabbit mAb	#2087	110	Rabbit	CST	WB 1:2000
Axin2(76G6) Rabbit mAb	#2151	95,98	Rabbit	CST	WB 1:2000
CyclinE1(D7T3U) Rabbit mAb	#20808	48	Rabbit	CST	WB 1:2000
β-catenin Antibody (Carboxy-terminal Antigen)	#9587	92	Rabbit	CST	WB 1:2000<br>IF 1:200
CyclinD1(DCS-6)	sc-20044	37	Mouse	Santa	WB 1:2000
Anti-GPCR GPR48 antibody(LGR4)	ab75501	104	Rabbit	Abcam	WB 1:2000<br>IP 1:100
c-Myc(9E10)	sc-40	67	Mouse	Santa	WB 1:2000
p44/42 MAPK(Erk1/2) Antibody	#9102	42,44	Rabbit	CST	WB 1:2000
PTEN(138G6) Rabbit mAb	#9559	54	Rabbit	CST	WB 1:2000
R-Spondin2(C-12)	sc-74883	28	Goat	Santa	WB 1:2000<br>IHC 1:200
Src(36D10) Rabbit mAb	#2109	60	Rabbit	CST	WB 1:2000<br>IF 1:400

					IP 1:100
Purified Mouse Anti-E-Cadherin	#610181	120	Mouse	BD	WB 1:2000
Purified Mouse Anti-N-Cadherin	#610920	130	Mouse	BD	WB 1:2000
GAPDH(14C10) Rabbit mAb	#2118	37	Rabbit	CST	WB 1:6000
p-EGFR(Tyr1068) (D7A5)	#3777	175	Rabbit	CST	WB 1:2000
EGFR	#2232	175	Rabbit	CST	WB 1:2000
Z0-1(H-300)	sc-10804	220	Mouse	BD	WB 1:2000
p-FAK(Tyr397)	#3283	125	Rabbit	BD	WB 1:2000
β-actin	#4970	45	Rabbit	CST	WB 1:2000
FAK	#71433	125	Rabbit	CST	WB 1:2000
MMP7	ab39984	30	Goat	abcam	WB 1:2000
MMP2	ab80737	74	Mouse	abcam	WB 1:2000
LGR5	AP2745d	100	Rabbit	ABGENT	WB 1:2000
Integrin αv	sc-9969	125-135	Mouse	Santa	WB 1:2000<br>IP 1:100<br>IF 1:200
Integrin α4	sc-365209	150	Mouse	Santa	WB 1:2000
Integrin α5	sc-376199	150	Mouse	Santa	WB 1:2000
Integrin β1	sc-374429	138	Mouse	Santa	WB 1:2000
Integrin β3	sc-365679	125	Mouse	Santa	WB 1:2000<br>IP 1:100<br>IF 1:200
HA Epitope Tag Antibody	NB600-363		Rabbit	Novus Biologicals	WB 1:2000
Myc-tag Rabbit mAb	#2278		Rabbit	CST	WB 1:2000<br>IF 1:200
Myc-tag Mouse mAb	#2276		Mouse	CST	WB 1:2000<br>IF 1:200

DYKDDDDK Tag (FLAG)	#8146		Mouse	CST	WB 1:2000
His-tag	#12698		Rabbit	CST	WB 1:2000
Fibronectin	610077	240	Mouse	BD transduction laboratories	WB 1:2000
Normal Rabbit IgG	#2729		Rabbit	CST	IP 1:100
Mouse mAb IgG1 Isotype Control	#5415		Mouse	CST	IP 1:100
LAMP1	sc-20011	120	Mouse	Santa	IF 1:200
Frizzled6 (D16E5) Rabbit mAb	#5158	80	Rabbit	CST	WB 1:1000
Anti-Frizzled 7 antibody	ab64636	90	Rabbit	Abcam	WB 1:1000

Table S3. Primers used for cloning and qRT-PCR (Related to Figure 1-6)..

**Gene**	**Forward primer (5'-3')**	**Reverse primer (5'-3')**	**Application**
RSPO2	ACAATACTGTGTCCAACCAT	TCCTCTTCTCCTTCGCCTTT	qRT-PCR
LGR4	GCTGTGCATTTTGGGGTTGTG	AATTTGGACGAAGGCAGTGATGTA	qRT-PCR
GAPDH	ACGGATTTGGTCGTATTGGGC	CTCGCTCCTGGAAGATGGTGAT	qRT-PCR
RSPO2	CCGGATCCGCCACCATGCAGTTTCGCCTTTTCTCCT	CCCAAGCTTCTCGAGTTATTGGTTAGCTCTGTCTGT	Cloning
FUs	CCGGATCCGCCACCATGCAGTTTCGCCTTTTCTCCT	CCCTCGAGTTCACATCCTTCCACACATT	Cloning
TSP	CCCTCGAGTTGGTTAGCTCTGTCTGTAGCT	CCGGATCCGCCACCATGTGTGAAGTTGGTCATTGGAG	Cloning

NOTE: qRT-PCR, quantitative real-time PCR

Figure S1

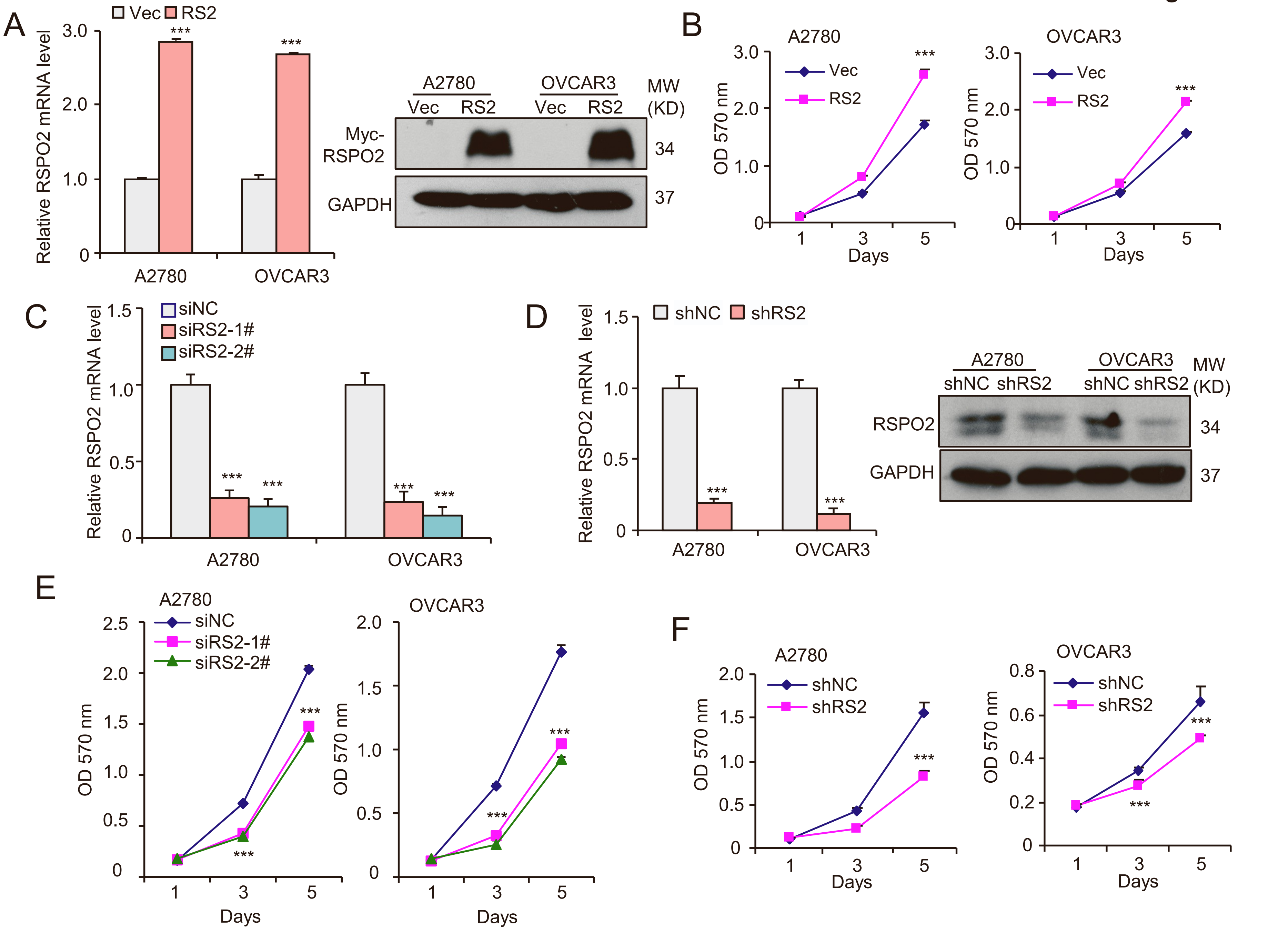
A
Vec RS2
Relative RSPO2 mRNA level
3.0
2.0
1.0
0
***
A2780
OVCAR3
A2780
OVCAR3
Vec RS2 Vec RS2
MW (KD)
Myc-RSPO2
GAPDH
34
37
B
A2780
OVCAR3
Vec
RS2
OD 570 nm
Days
1 3 5
C
siNC
siRS2-1#
siRS2-2#
Relative RSPO2 mRNA level
1.5
1.0
0.5
D
shNC shRS2
RSPO2
E
siNC
siRS2-1#
siRS2-2#
2.5
F
shNC
shRS2
0.8
0.6
0.4
0.2

Figure S2

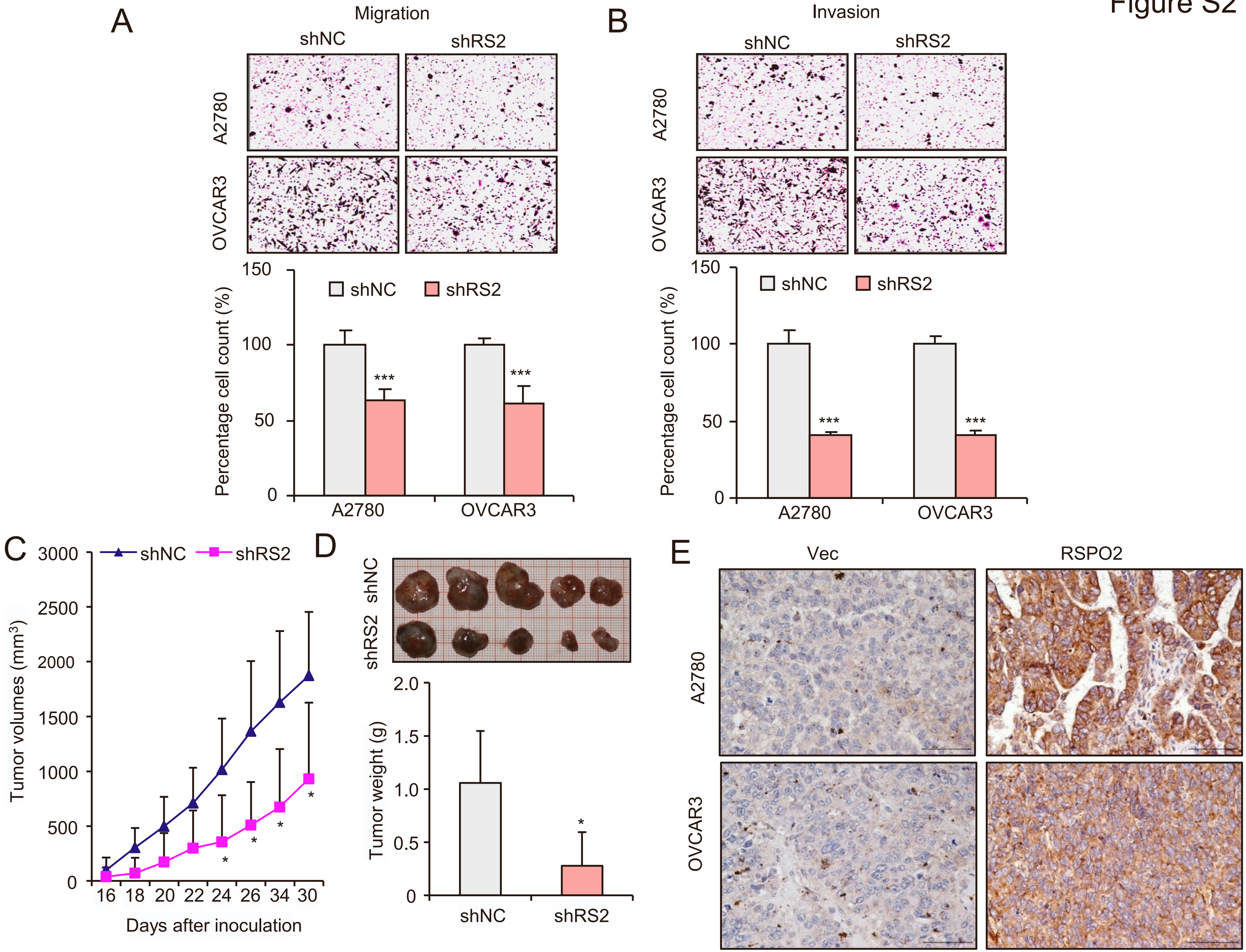

A
Migration
shNC
shRS2
A2780
OVCAR3
150
100
50
0
Percentage cell count (%)
shNC
shRS2
***
***
A2780
OVCAR3
B
Invasion
shNC
shRS2
A2780
OVCAR3
150
100
50
0
Percentage cell count (%)
shNC
shRS2
***
***
A2780
OVCAR3
C
3000
2500
2000
1500
1000
500
0
shNC
shRS2
Tumor volumes (mm3)
*
*
*
*
16
18
20
22
24
26
34
30
Days after inoculation
D
shNC
shRS2
2.0
1.5
1.0
0.5
0
Tumor weight (g)
*
shNC
shRS2
E
Vec
RSPO2
A2780
OVCAR3

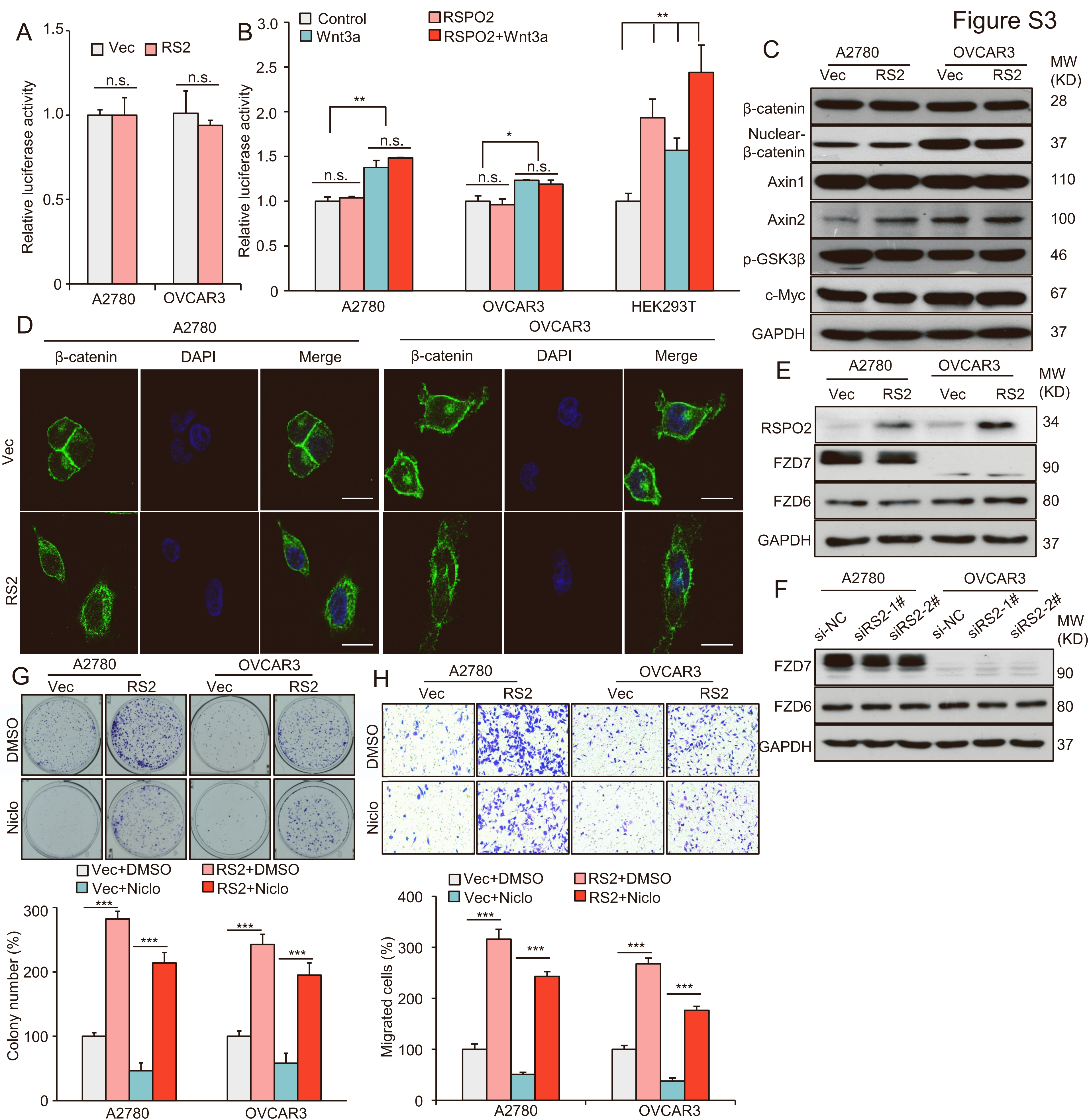

Figure S3
A
Vec
RS2
n.s.
n.s.
Relative luciferase activity
A2780
OVCAR3
B
Control
Wnt3a
RSPO2
RSPO2+Wnt3a
Relative luciferase activity
A2780
OVCAR3
HEK293T
C
A2780
OVCAR3
Vec
RS2
MW (KD)
β-catenin
Nuclear-β-catenin
Axin1
Axin2
p-GSK3β
c-Myc
GAPDH
D
β-catenin
DAPI
Merge
E
RSPO2
FZD7
FZD6
F
si-NC
siRS2-1#
siRS2-2#
G
DMSO
Niclo
Vec+DMSO
Vec+Niclo
RS2+DMSO
RS2+Niclo
Colony number (%)
H
Migrated cells (%)

Figure S4

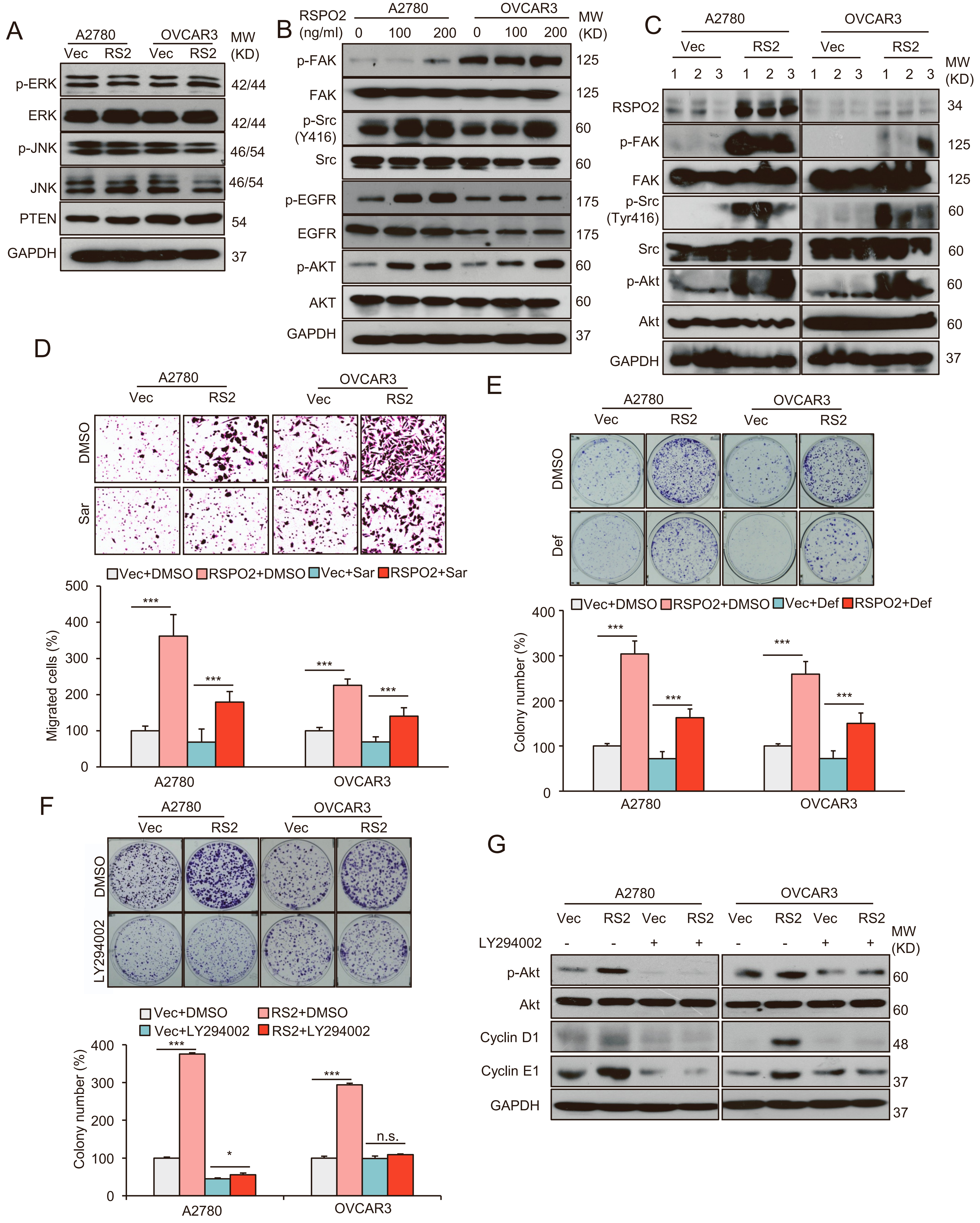

A
A2780
OVCAR3
Vec
RS2
Vec
RS2
MW (KD)
p-ERK
ERK
p-JNK
JNK
PTEN
GAPDH
42/44
42/44
46/54
46/54
54
37
B
RSPO2 (ng/ml)
A2780
OVCAR3
0 100 200 0 100 200
MW (KD)
p-FAK
FAK
p-Src (Y416)
Src
p-EGFR
EGFR
p-AKT
AKT
GAPDH
125
125
60
60
175
175
60
60
37
C
A2780
OVCAR3
Vec
RS2
Vec
RS2
1 2 3 1 2 3 1 2 3 1 2 3
MW (KD)
RSPO2
p-FAK
FAK
p-Src (Tyr416)
Src
p-Akt
Akt
GAPDH
34
125
125
60
60
60
60
37
D
A2780
OVCAR3
Vec
RS2
Vec
RS2
DMSO
Sar
Vec+DMSO
RSPO2+DMSO
Vec+Sar
RSPO2+Sar
Migrated cells (%)
0
100
200
300
400
500
***
***
***
***
A2780
OVCAR3
E
A2780
OVCAR3
Vec
RS2
Vec
RS2
DMSO
Def
Vec+DMSO
RSPO2+DMSO
Vec+Def
RSPO2+Def
Colony number (%)
0
100
200
300
400
***
***
***
***
A2780
OVCAR3
F
A2780
OVCAR3
Vec
RS2
Vec
RS2
DMSO
LY294002
Vec+DMSO
RS2+DMSO
Vec+LY294002
RS2+LY294002
Colony number (%)
0
100
200
300
400
***
*
***
n.s.
A2780
OVCAR3
G
A2780
OVCAR3
Vec RS2 Vec RS2 Vec RS2 Vec RS2
LY294002 - - + + - - + +
MW (KD)
p-Akt
Akt
Cyclin D1
Cyclin E1
GAPDH
60
60
48
37
37

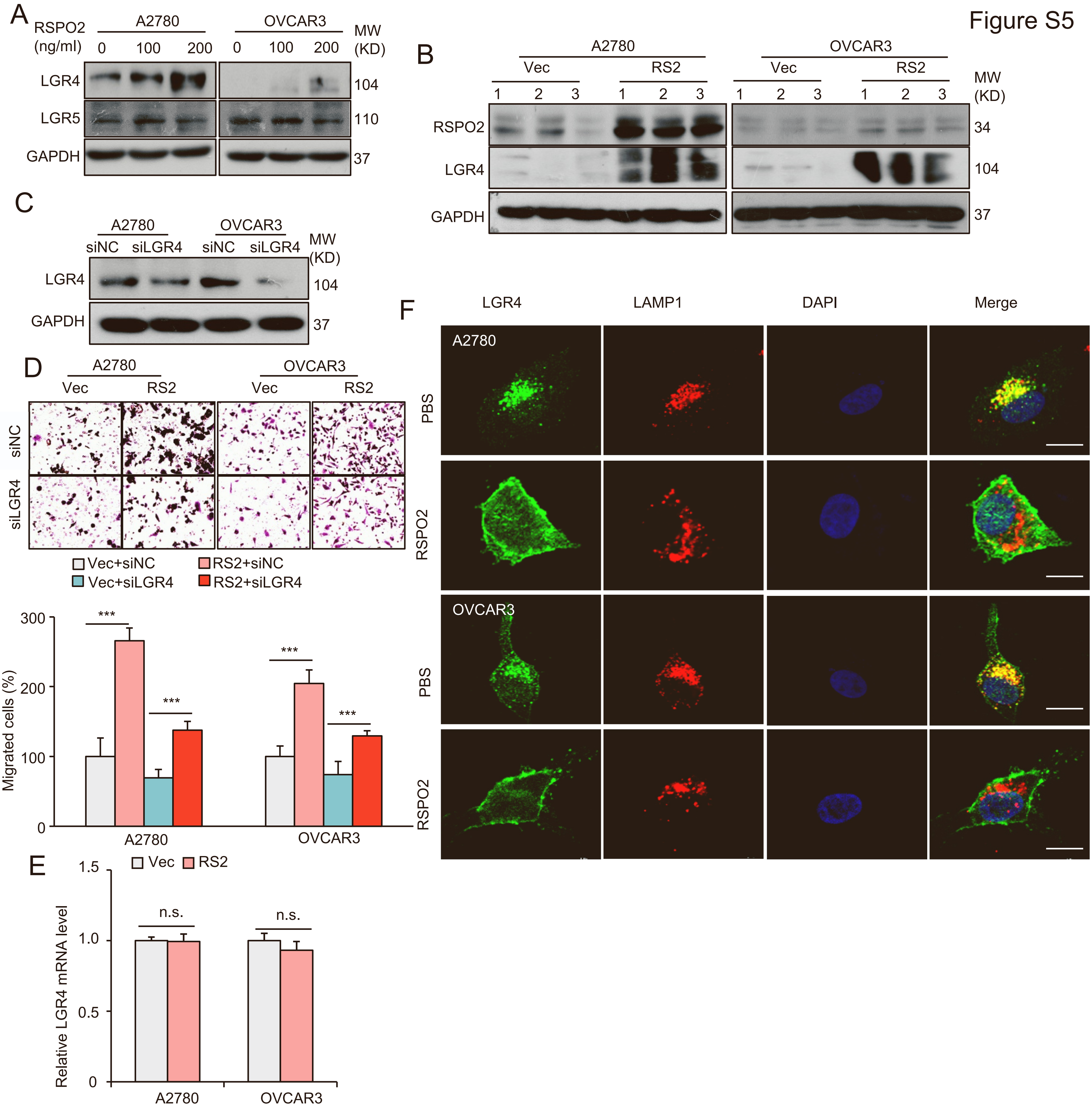
Figure S5
A
RSPO2 (ng/ml)
A2780
OVCAR3
0 100 200 0 100 200
MW (KD)
LGR4
LGR5
GAPDH
104
110
37
B
Vec
RS2
1 2 3
RSPO2
34
C
siNC siLGR4
D
siNC
siLGR4
Vec+siNC
RS2+siNC
Vec+siLGR4
RS2+siLGR4
Migrated cells (%)
300
200
100
0
***
E
Vec RS2
Relative LGR4 mRNA level
1.5
1.0
0.5
n.s.
F
LAMP1
DAPI
Merge
PBS

Figure S6

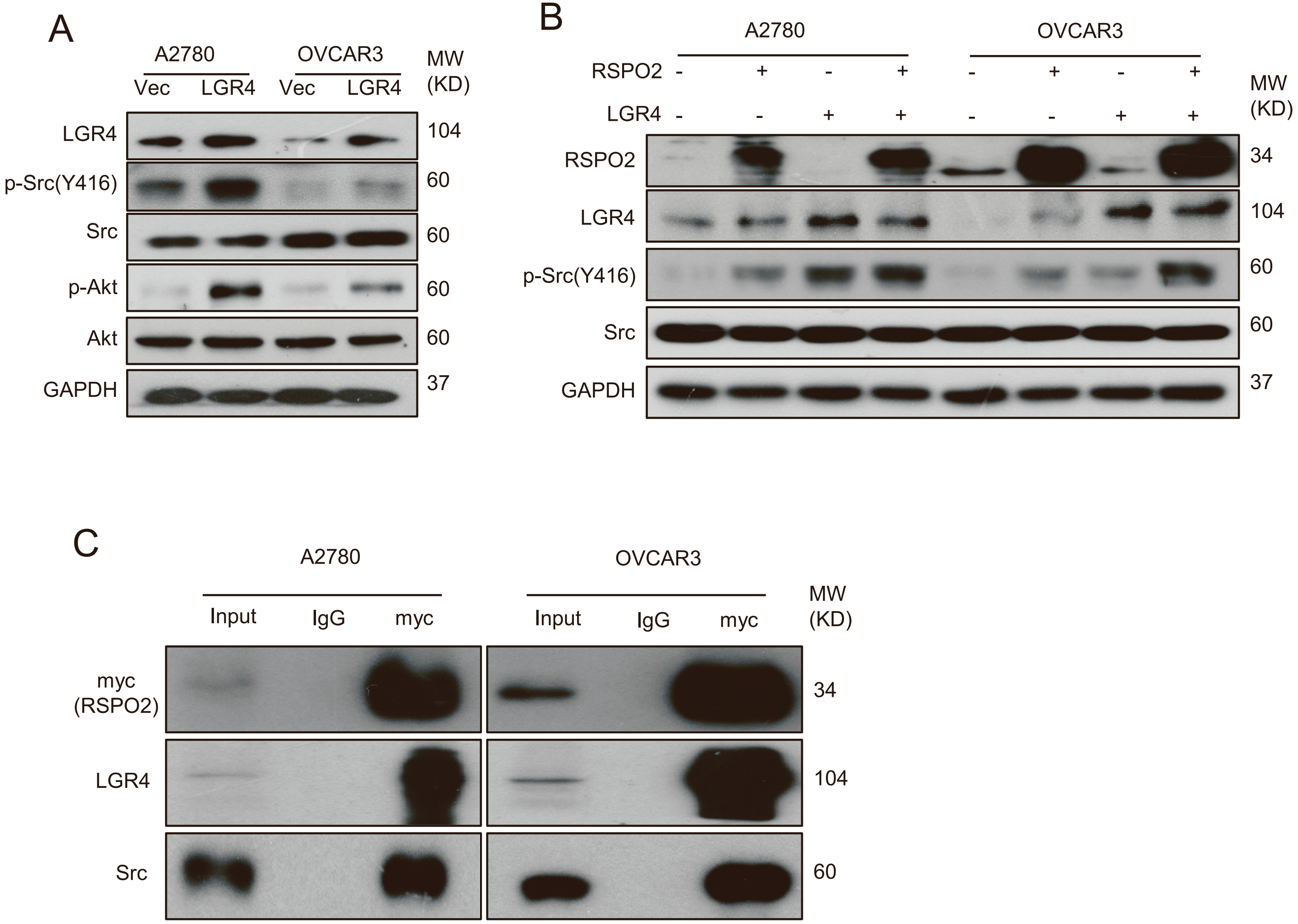
A
A2780
OVCAR3
Vec
LGR4
Vec
LGR4
MW (KD)
LGR4
p-Src(Y416)
Src
p-Akt
Akt
GAPDH
104
60
60
60
60
37
B
A2780
OVCAR3
RSPO2
LGR4
RSPO2
LGR4
p-Src(Y416)
Src
GAPDH
MW (KD)
34
104
60
60
37
C
A2780
OVCAR3
Input
IgG
myc
Input
IgG
myc
MW (KD)
myc (RSPO2)
LGR4
Src
34
104
60

Supplementary Fig. 7

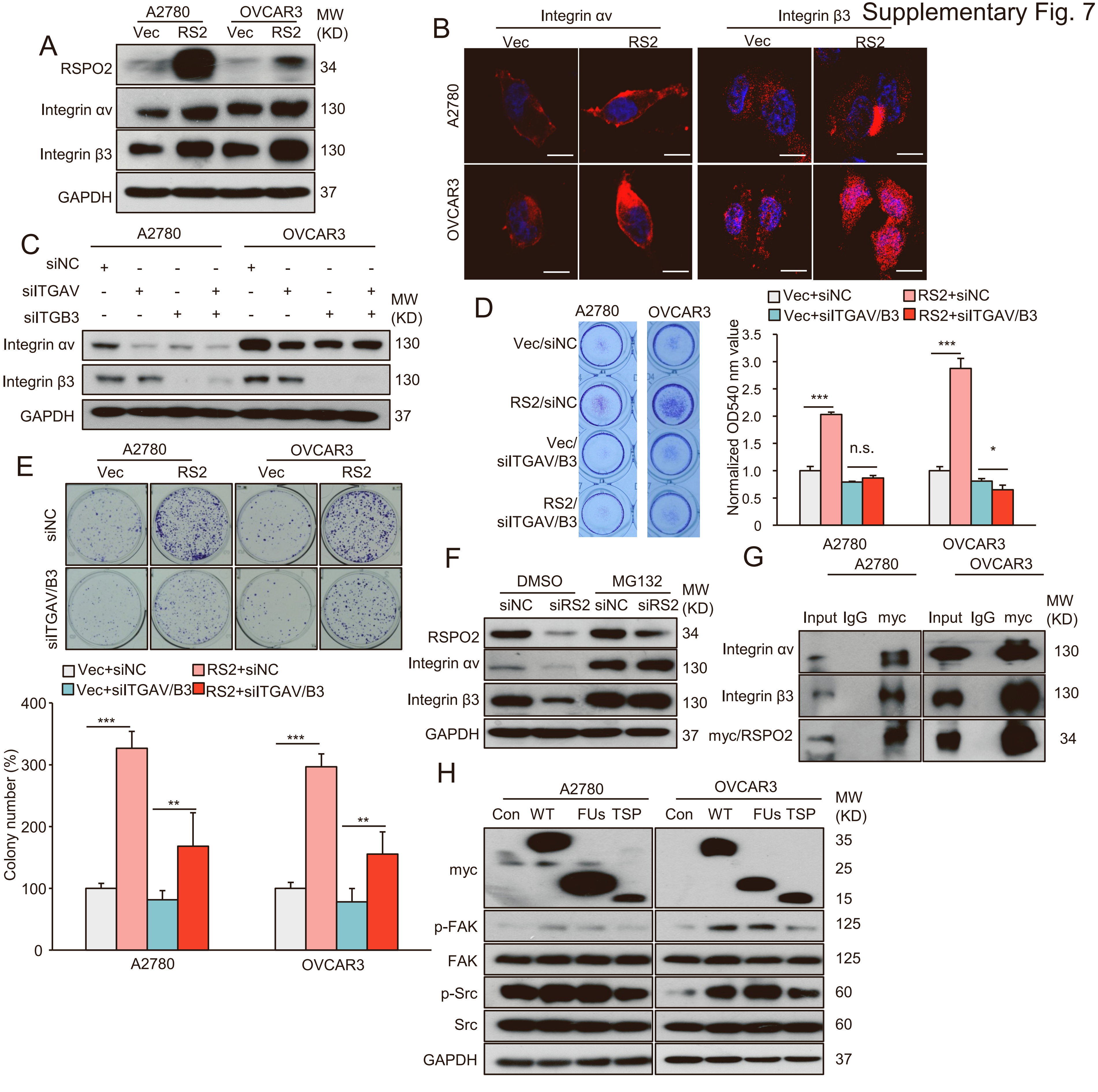

Supplement: Document S1. Figures S1–S7 and Tables S1–S3 [file mmc1.pdf]
